# Supplementary material for: Growth in fluctuating light buffers plants against photorespiratory perturbations
Source: Nat Commun. 2023 Nov 3;14:7052. doi: 10.1038/s41467-023-42648-x (PMC10624928; doi:10.1038/s41467-023-42648-x)
Supplement: Supplementary file 1 — Supplementary Information [file 41467_2023_42648_MOESM1_ESM.pdf]

Supplementary information

Growth in fluctuating light buffers plants against photorespiratory perturbations

Thekla von Bismarck[1,2,3*], Philipp Wendering[3,4], Leonardo Perez de Souza[3], Jeremy Ruß[3], Linnéa Strandberg[3], Elmien Heyneke[3], Berkley J. Walker[5,6], Mark A. Schöttler[3], Alisdair R. Fernie[3], Zoran Nikoloski[3,4], Ute Armbruster[1,2,3*]

[1]Molecular Photosynthesis, Heinrich-Heine-University Düsseldorf, Universitätsstraße 1, 40225 Düsseldorf, Germany

[2]CEPLAS - Cluster of Excellence on Plant Sciences, Heinrich Heine University Düsseldorf, Düsseldorf, Germany

[3]Max Planck Institute of Molecular Plant Physiology, Am Mühlenberg 1, 14476 Potsdam

[4]Bioinformatics Department, Institute of Biochemistry and Biology, University of Potsdam, Karl-Liebknecht-Str. 24-25, 14476 Potsdam

[5]DOE-Plant Research Laboratory, Michigan State University, 612 Wilson Rd, East Lansing, MI 48824, USA

[6]Department of Biochemistry and Molecular Biology, Michigan State University, 603 Wilson Rd Rm 212, East Lansing, MI 48823, USA

*Corresponding author e-mails: thekla@v-bismarck.de, ute.armbruster@hhu.de

1. Supplementary figures

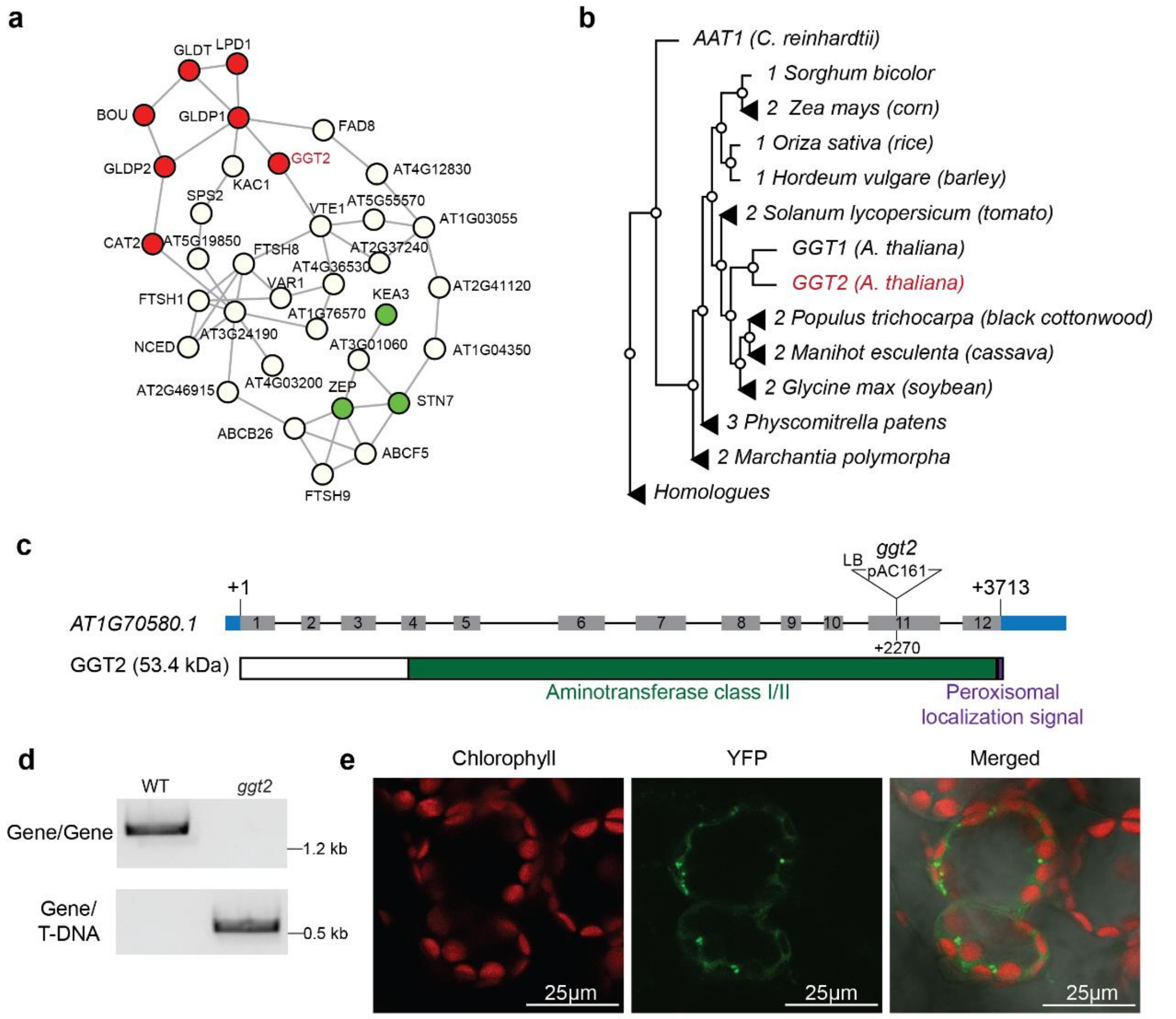

Supplementary Figure 1. Co-expression network including *GGT2*, phylogenetic analysis, *ggt2* mutant information and localization.

a. Co-expression network based on *KEA3* (*At4g04850*), *ZEP* (*At5g67030*) and *STN7* (*At1g68830*) in green using ATTEDII (https://atted.jp/)[1] and Cytoscape setting from this website for graphical representation, includes multiple genes involved in photorespiration and GGT2 in red. **b.** Phylogenetic analysis using the Phytozome website (phytozome-next.jgi.doe.gov) points towards independent duplication events of a GGT gene in most plant species. **c.** Gene and protein model of GGT2 with the T-DNA insertion of *ggt2* being located in the 11th intron. Grey boxes indicate exons connected by introns (black line) and are up to scale, and blue boxes 5' and 3' untranslated regions. **d.** PCR on genomic DNA revealing the integration of the Gabi-Kat T-DNA in the *ggt2* line. The upper panel shows the amplification product using gene-specific primers spanning the T-DNA insertion site (Gene/Gene) with the sequences 5'-CTGGCCAGTGTCTTAGCGA-3' (forward) and 5'-GAGTTTGACACAGAGTAGGACCA-3' (reverse) and the lower panel the amplification product using the left border primer of the T-DNA with the sequence 5'-ATAATAACGCTGCGGACATCTACATTTT-3' and the forward gene-specific primer (Gene/T-DNA). **e.** Fluorescence microscopy pictures revealing the subcellular localization of GGT2-YFP. The left panel shows chlorophyll fluorescence in red, the middle panel YFP-fluorescence in green and the right panel a merged image of both channels together with a bright-field image.

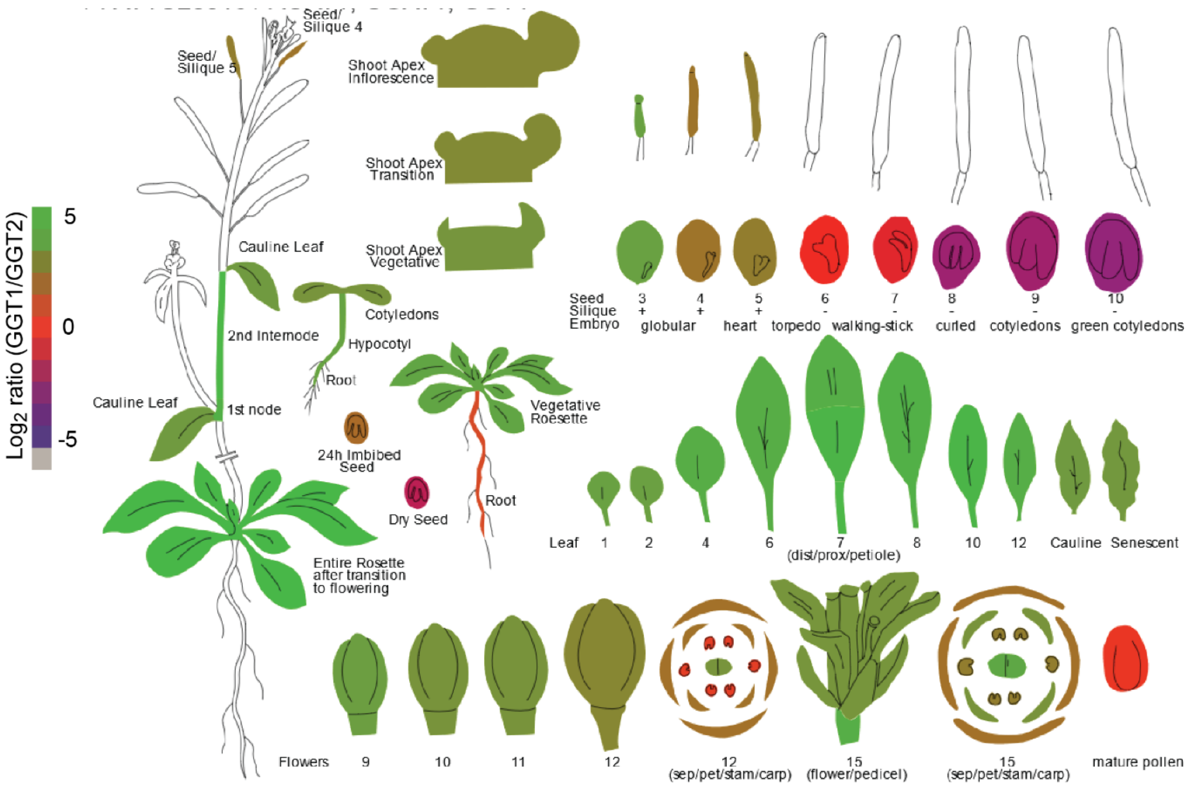

Supplementary Figure 2. Expression pattern of *GGT1* and *GGT2.*
Expression levels of *GGT1* relative to *GGT2* in different organs of *Arabidopsis thaliana* plants using the Arabidopsis eFP Browser 2.0 (http://bar.utoronto.ca)[2].

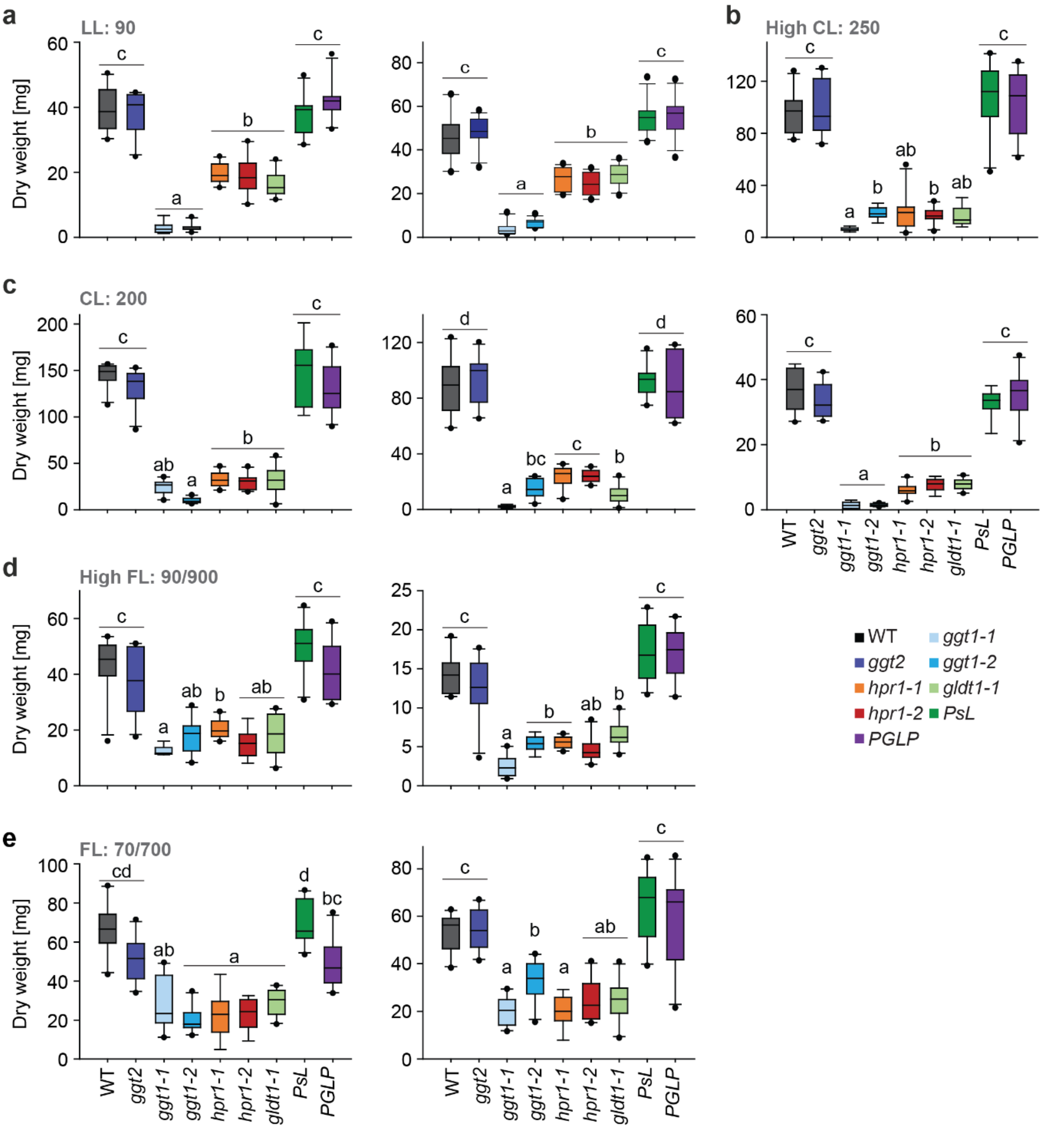

Supplementary Figure 3. Different growth experiments at various fluctuating and non-fluctuating light conditions.

a-e. WT, *ggt2,* two mutant alleles of each *ggt1* and *hpr1, gldt1-1, PsL* and *PGLP* were grown under non-fluctuating (a-c) or fluctuating light (d-e) with different light intensities: 90 µmol photons m^{-2} s^{-1} for 36 (left) or 37 (right) days after sowing (d.a.s.; a), 250 µmol photons m^{-2} s^{-1} for 31 d.a.s. (b), 200 µmol photons m^{-2} s^{-1} for 36 (left panel), 34 d.a.s. (middle panel) or 29 d.a.s. (right panel; c), 1 min 900 µmol photons m^{-2} s^{-1} and 4 min 90 µmol photons m^{-2} s^{-1} for 38 (left panel) and 31 d.a.s. (right panel; d), and two independent experiments each at 1 min 700 µmol photons m^{-2} s^{-1} and 4 min 70 µmol photons m^{-2} s^{-1} for 43 d.a.s. (e). Boxplots represent n = 7 (b: *ggt1-1*; c, right: *ggt1-1*), n = 8 (c, middle: *ggt1-1*), n = 9 (a, left: *ggt1-1*; b: *ggt1-2*, *gldt1-1*; c, left: *PsL*; c, right: *hpr1-2*, *PsL*; d, left: *ggt1-1*, *hpr1-2*; d, right: *ggt1-2*; e, left: *hpr1-1*, *hpr1-2*; e, right: *hpr1-1*), n = 11 (a, right: *hpr1-1*), n = 12 (a, right: WT, *ggt2*, *ggt1-2*, *hpr1-2*, *gldt1-1*, *PsL*, *PGLP*) and n = 10 for all remaining. The lower and upper boundaries of the box indicate the 25th and 75th percentile, the median is shown by the middle line. Whiskers indicate maximum and minimum and black circles represent outliers that fell outside the 10th and 90th percentiles. Different lowercase letters above boxes indicate significant differences between groups within one experiment as determined by two-sided Games-Howell multiple comparison with $p < 0.05$.

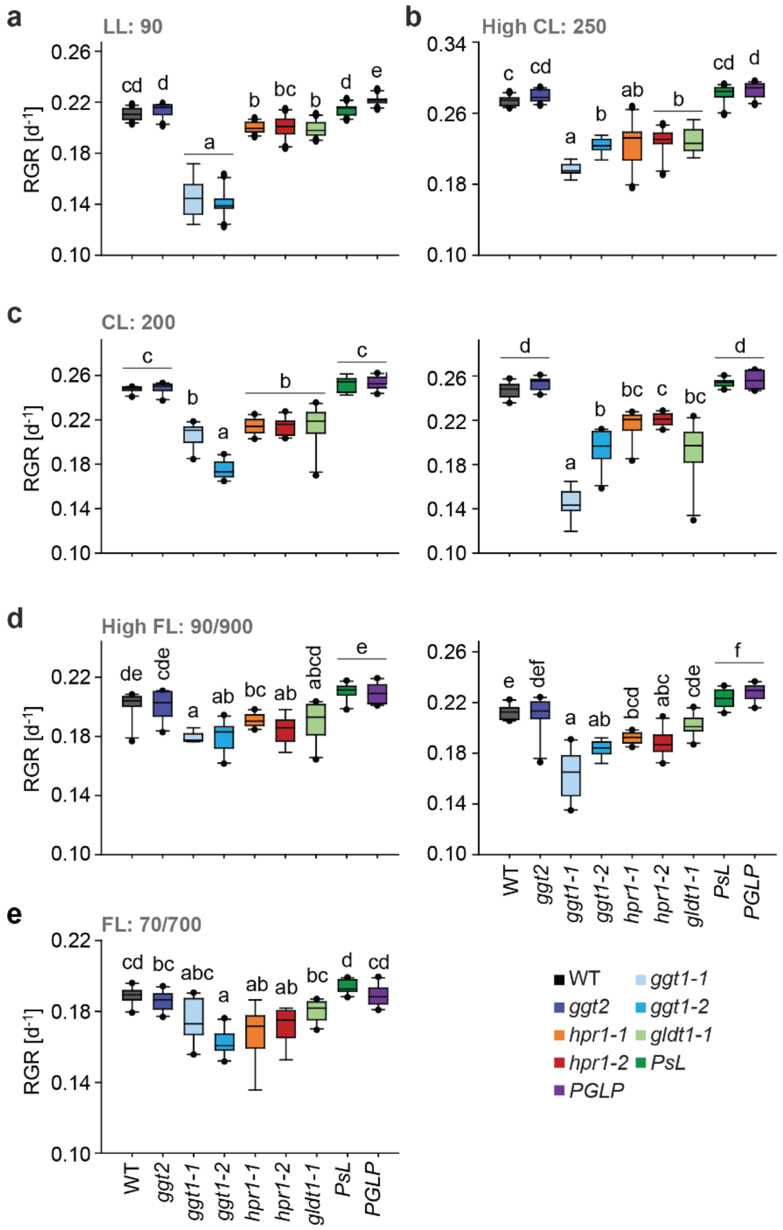

Supplementary Figure 4. Relative growth rates in various fluctuating and non-fluctuating light conditions.

a-e. WT, *ggt2,* two mutant alleles of each *ggt1* and *hpr1, gldt1-1, PsL* and *PGLP* were grown under non-fluctuating (a-c) or fluctuating light (d-e) with different light intensities: 90 µmol photons m^{-2} s^{-1} for 36 (left) or 37 (right) days after sowing (d.a.s.; a), 250 µmol photons m^{-2} s^{-1} for 31 d.a.s. (b), 200 µmol photons m^{-2} s^{-1} for 36 (left panel), 34 d.a.s. (middle panel) or 29 d.a.s. (right panel; c), 1 min 900 µmol photons m^{-2} s^{-1} and 4 min 90 µmol photons m^{-2} s^{-1} for 38 (left panel) and 31 d.a.s. (right panel; d), 1 min 700 µmol photons m^{-2} s^{-1} and 4 min 70 µmol photons m^{-2} s^{-1} both for 43 d.a.s. (e). Relative growth rates (RGR), i.e., the rate of accumulation of new dry mass per unit of existing dry mass per day, were calculated from dry weight in Supplementary Figure 3. Boxplots represent n = 7 (b: *ggt1-1*), n = 8 (c, right: *ggt1-1*), n = 9 (a: *ggt1-1*; b: *ggt1-2*, *gldt1-1*; c, left: *PsL*; d, left: *ggt1-1*, *hpr1-2*; d, right: *ggt1-2*; e: *hpr1-1*, *hpr1-2*; e, right: *hpr1-1*) and n = 10 for all remaining. The lower and upper boundaries of the box indicate the 25th and 75th percentile, the median is shown by the middle line. Whiskers indicate maximum and minimum and black circles represent outliers that fell outside the 10th and 90th percentiles. Different lowercase letters above boxes indicate significant difference between groups within one experiment as determined by two-sided Games-Howell multiple comparison with $p < 0.05$.

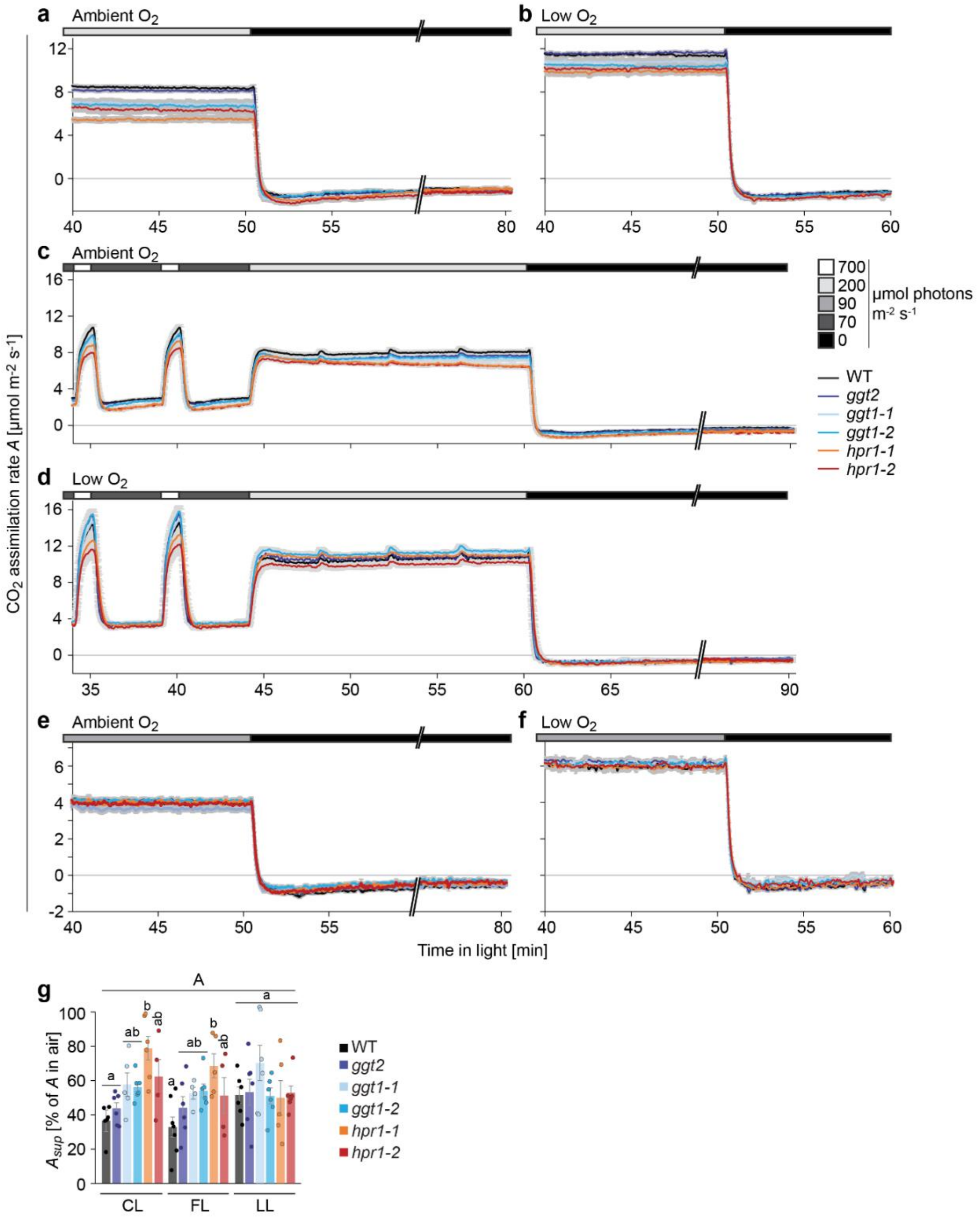

Supplementary Figure 5. CO_2 assimilation rate under ambient and low oxygen and relative oxygen-suppressed CO_2 assimilation.

a-f. WT, *ggt2* and two mutant alleles of each *ggt1* and *hpr1* were acclimated to CL (a-b), FL (c-d) or LL (e-f) as described in Fig. 2 and CO_2 assimilation rate *A* was determined for 45-50 min in growth light with air (21% O_2; a, c, e) or low oxygen (2% O_2; b, d, f). Subsequently, FL-grown plants were exposed to 15 min CL and all plants to a final 10-30 min of darkness. Note that maximal and minimal values of dynamic changes in panels c-d may be underestimated due to long latency times inside the IRGA. **g.** Relative O_2-suppressed CO_2 assimilation rate (A_{sup}) was calculated from data in Fig. 2b. Capital letters indicate significant differences between light conditions and lower-case letters between genotypes within one light condition as determined via two-way ANOVA and post-hoc Tukey multiple comparison test with $p < 0.05$. **a-g.** Averages of n = 4 (CL: *hpr1-2*; FL: *ggt1-1*, *hpr1-2*), n = 5 (CL: *ggt1-1*; FL: *hpr1-1*; LL: *hpr1-1*), n = 7 (FL: WT) and n = 6 for all remaining groups ± standard error (a-e) or standard deviation (g) are shown.

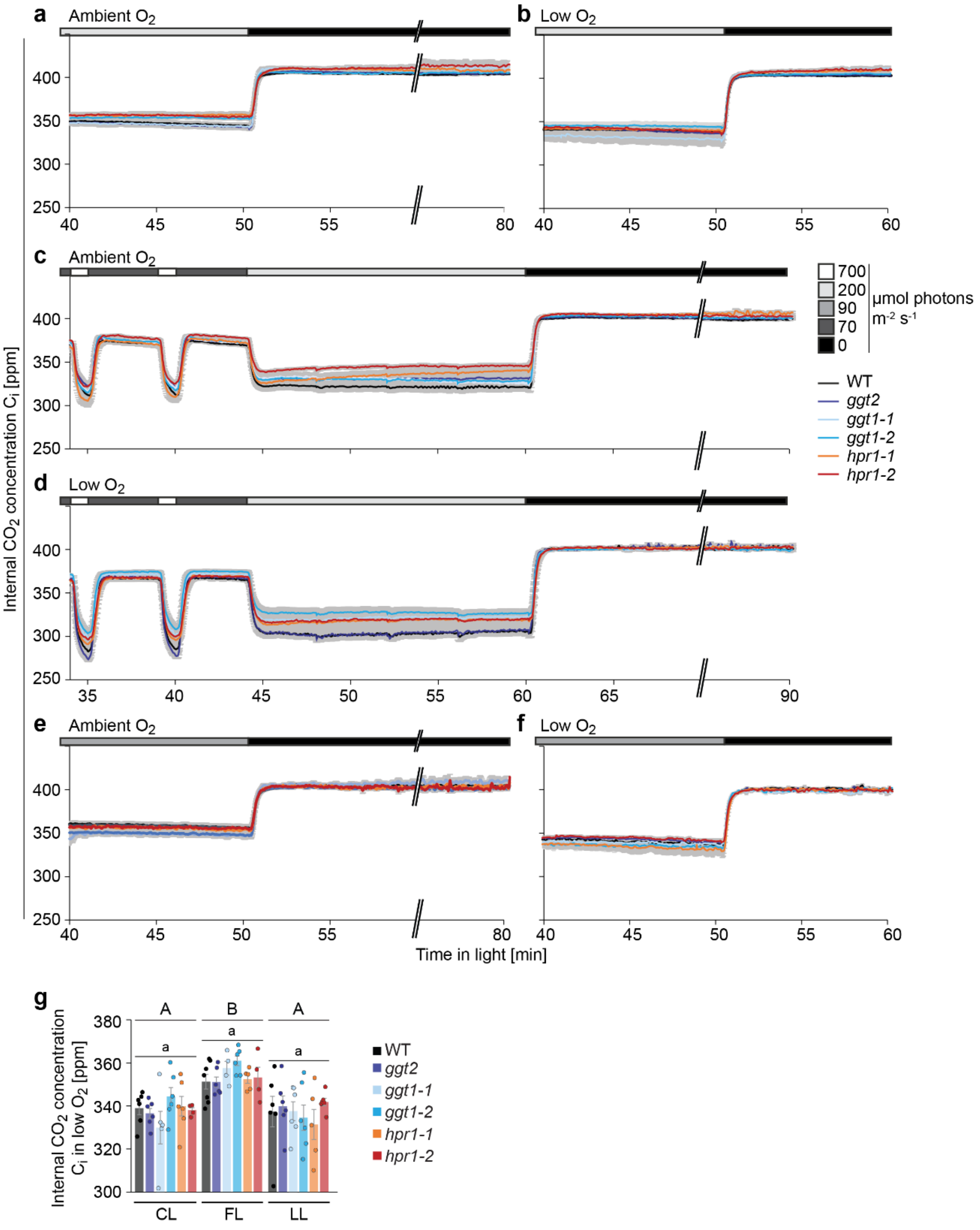

Supplementary Figure 6. Internal CO_2 concentration C_i under ambient and low oxygen. a-f. WT, *ggt2* and two mutant alleles of each *ggt1* and *hpr1* were acclimated to CL (a-b), FL (c-d) or LL (e-f) as described in Fig. 2 and internal CO_2 concentration C_i was determined for 45-50 min in growth light with air (21% O_2; a, c, e) or low oxygen (2% O_2; b, d, f). Subsequently, FL-grown plants were exposed to 15 min CL and all plants to a final 10-30 min of darkness. Note that maximal and minimal values of dynamic changes in panels c-d may be underestimated due to long latency times inside the IRGA. **g.** Average C_i in low O_2 was determined from data as in panels b, d and f averaged over the last two light fluctuations (FL, d) or the final 1.5 min at the end of the measurement (CL and LL; b and f). Capital letters indicate significant differences between light conditions and lower-case letters between genotypes within one light condition as determined via two-way ANOVA and post-hoc Tukey multiple comparison test with $p < 0.05$. **a-g.** Averages of n = 4 (CL: *hpr1-2*; FL: *ggt1-1*, *hpr1-2*), n = 5 (CL: *ggt1-1*; FL: *hpr1-1*; LL: *hpr1-1*), n = 7 (FL: WT) and n = 6 for all remaining groups ± standard error (a-e) or standard deviation (g) are shown.

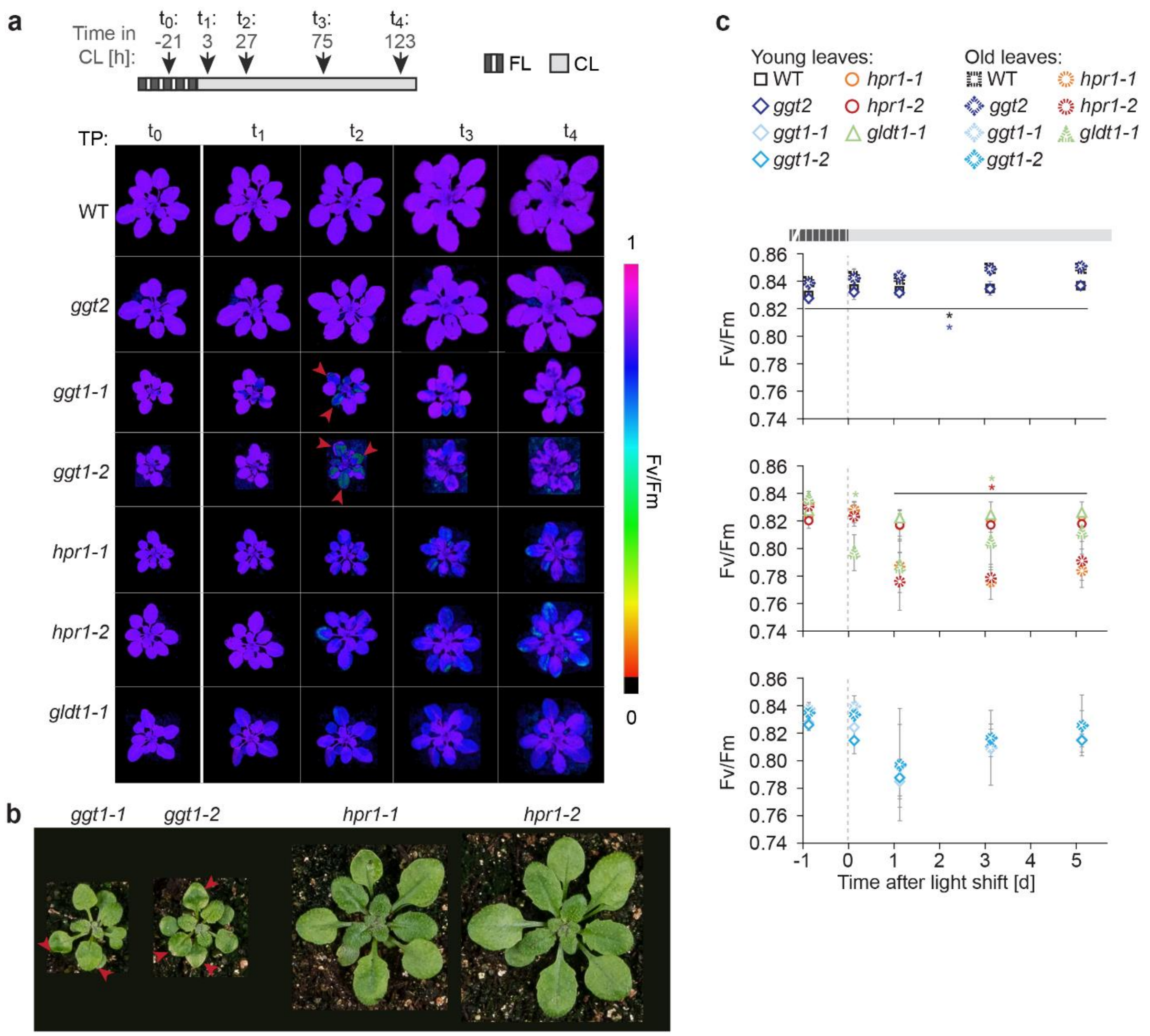

Supplementary Figure 7. Leaf age dependency of PSII function in different photorespiratory mutants and leaf appearance after a shift from fluctuating to control light.

a. Representative false color images of maximum quantum yield of PSII (F_v/F_m) of WT, *ggt2*, two mutant alleles of each *ggt1* and *hpr1* and *gldt1-1* under fluctuating light (FL: 1 min 700 µmol photons m^{-2} s^{-1}, 4 min 70 µmol photons m^{-2} s^{-1}) and 3 h, 27 h, 75 h and 123 h after the shift to control light (CL: 200 µmol photons m^{-2} s^{-1}). Per genotype and time point, 7-11 images (see below for exact n) were taken with similar results. **b,** Plant pictures of CL-grown *ggt1* and *hpr1* alleles magnified five-fold compared with Fig. 1b. **a-b.** Red arrows indicate low F_v/F_m (a) and chlorotic patches (b) found in the *ggt1* mutant only. **c.** F_v/F_m was calculated separately from old and young leaves, defined as leaves with and without visible petiole, respectively. Genotypes separated into three groups: WT and *ggt2* increased F_v/F_m slightly after the light shift and young leaves had significantly lower F_v/F_m as compared to old leaves (upper graph); *gldt1-1* and *hpr1* lines decreased F_v/F_m upon a shift to CL, with a more pronounced effect in old compared with young leaves (middle graph). *ggt1* lines decreased F_v/F_m upon a shift to CL without discernable differences between young and old leaves (bottom graph). Averages of n = 10 for all genotypes except for n = 7 for *hpr1-1* (t_4), n = 8 for *hpr1-2* (t_4), n = 9 for *hpr1-1* (t_0-t_3), *gldt1-1* (t_0-t_4) and *ggt1-2* (t_4) and n = 11 for *hpr1-2* (t_0-t_3) ± standard deviation are shown. Asterisks in the corresponding color indicate significant differences between young and old leaves at a given time point determined via two-way ANOVA and post-hoc Tukey multiple comparison test with $p < 0.05$. To exclude background signal due to algal growth on the soil, the outline of each plant was selected manually to generate the average F_v/F_m.

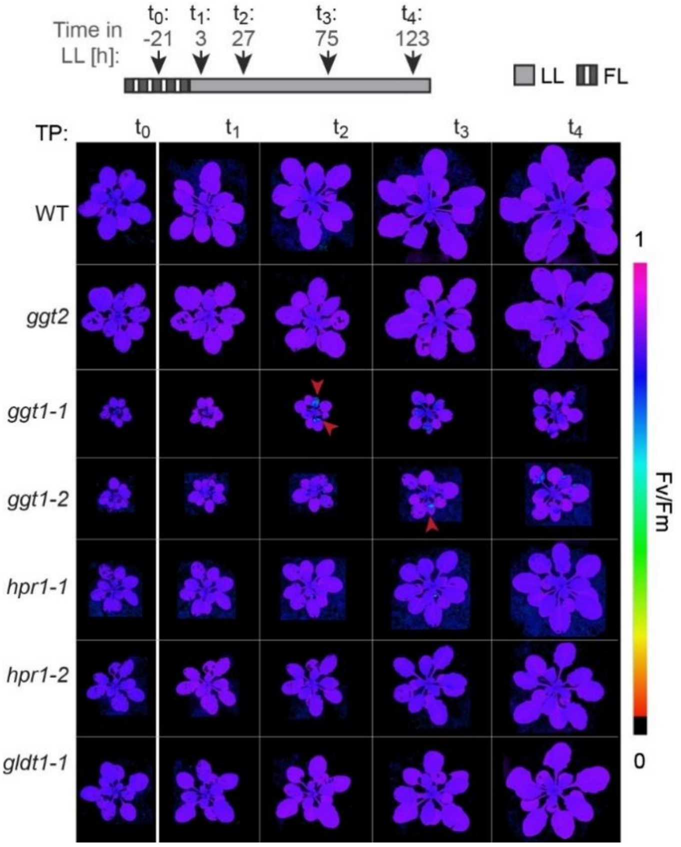

Supplementary Figure 8. Chlorophyll fluorescence images of F_v/F_m during a shift from fluctuating to low light.

Representative false color images of maximum quantum yield of PSII (F_v/F_m) of WT, *ggt2*, two mutant alleles of each *ggt1* and *hpr1* and *gldt1-1* grown under fluctuating light (FL: 1 min 700 µmol photons m^{-2} s^{-1}, 4 min 70 µmol photons m^{-2} s^{-1}; time point t_0) and 3 h (t_1), 27 h (t_2), 75 h (t_3) and 123 h (t_4) after the shift to low light (LL: 90 µmol photons m^{-2} s^{-1}) as in Fig. 3c-d. Red arrows point to patchy low F_v/F_m found in the *ggt1* mutant only. Per genotype and time point, 10 images with similar results were taken.

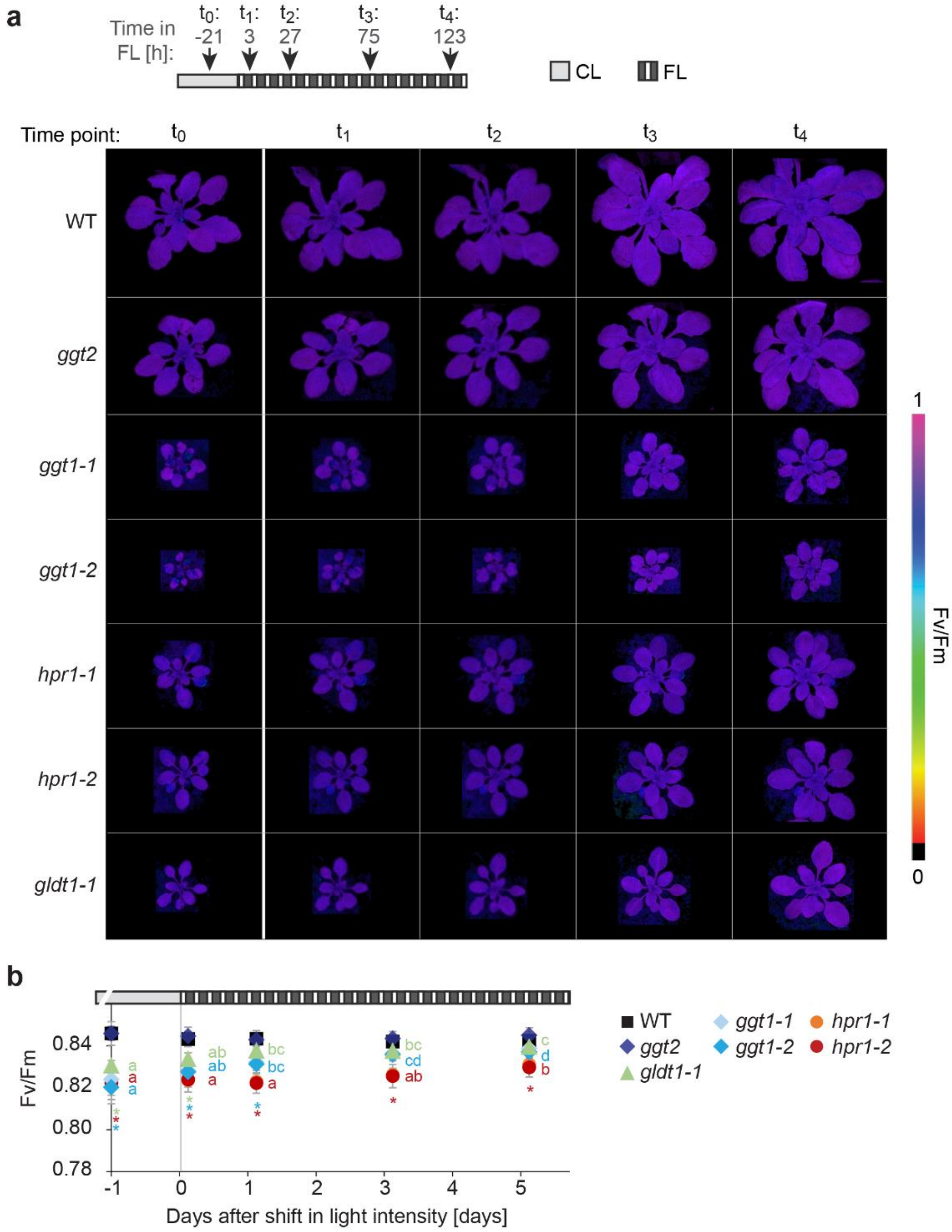

Supplementary Figure 9. F_v/F_m of photorespiratory mutants during a shift from control to fluctuating light.

a-b. Representative false color images (a) and average (b) maximum quantum yield of PSII (F_v/F_m) of WT, *ggt2*, two mutant alleles of each *ggt1* and *hpr1* and *gldt1-1* grown under control light (CL: 200 µmol photons m^{-2} s^{-1}; time point t_0) and 3 h (t_1), 27 h (t_2), 75 h (t_3) and 123 h (t_4) after the shift to fluctuating light (FL: 1 min 700 µmol photons m^{-2} s^{-1}, 4 min 70 µmol photons m^{-2} s^{-1}). Per genotype and time point, 10 images with similar results were taken. **b,** Averages of n = 10 ± standard deviation are shown. Asterisks in the corresponding color indicate significant differences between mutants and WT within one time point and different lowercase letters in the corresponding color significant differences between time points within one genotype determined via two-way ANOVA and post-hoc Tukey multiple comparison test with $p < 0.05$. To exclude background signal due to algal growth on the soil, the outline of each plant was selected manually to generate the average F_v/F_m.

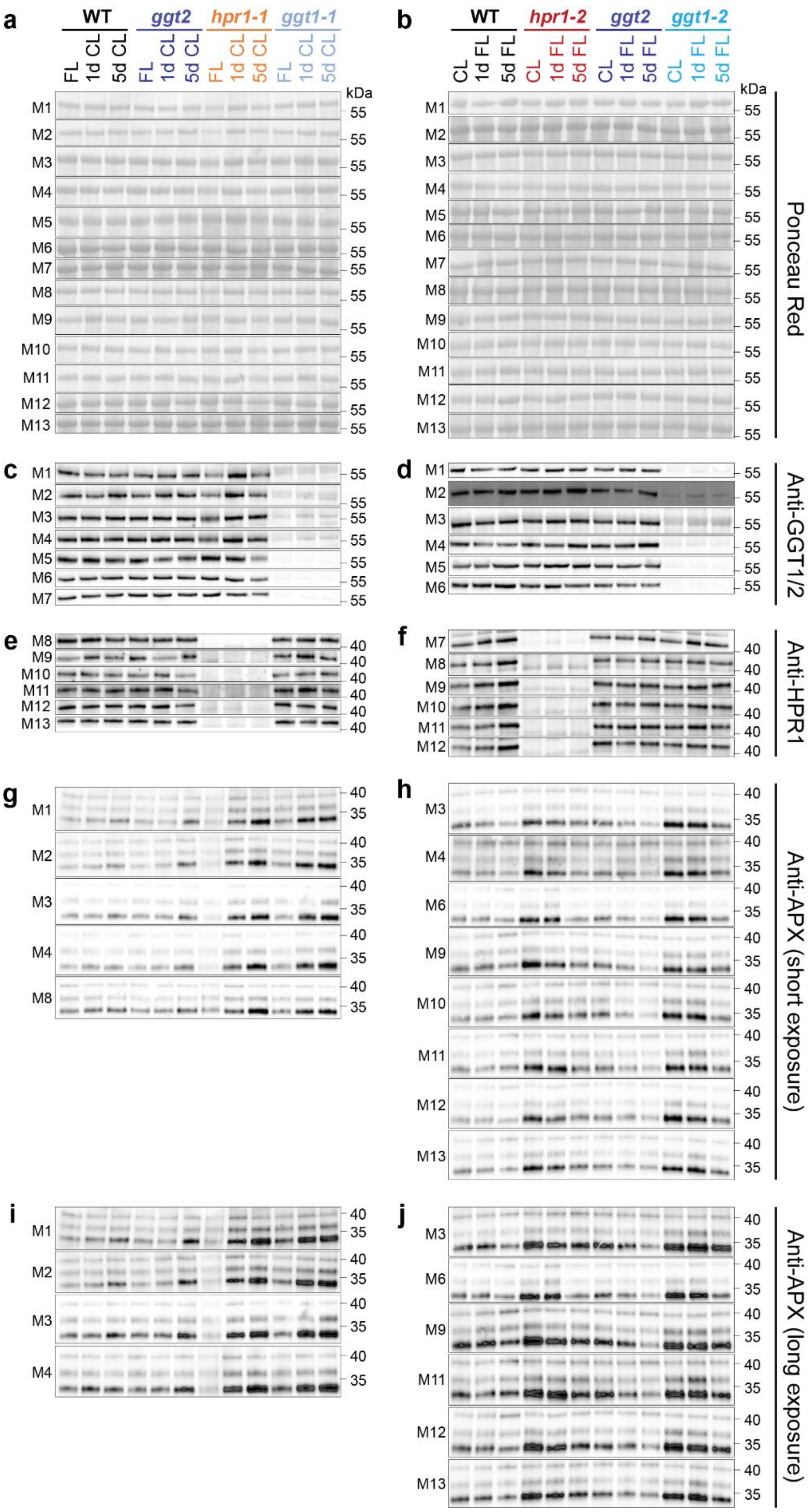

Supplementary Figure 10. Protein levels of GGT1/2, HPR1 and APX isoforms during light shifts.

a-j. Immunoblot analysis of total leaf protein extracts from WT, *ggt2*, *ggt1-1*, *ggt1-2*, *hpr1-1* and *hpr1-2* grown under fluctuating light (FL: 1 min 700 µmol photons m^{-2} s^{-1}, 4 min 70 µmol photons m^{-2} s^{-1}; 35 d) or control light (CL: 200 µmol photons m^{-2} s^{-1}; 29 d) and shifted to CL (a, c, e, g, i) or FL (b, d, f, h, j),

respectively. Samples were taken before the light shift (36 d in FL or 19 d in CL) and 27 h (1 d) and 123 h (5 d) after the light shift as in Fig. 3 and Supplementary Fig. 9. Membranes were stained with Ponceau Red (a-b) and subsequently incubated with anti-GGT1/2 (c-d), anti-HPR1 (e-f) and anti-ascorbate peroxidase (APX; g-j). Anti-GGT1/2 binds both GGT1 and GGT2. Anti-APX recognizes all four isoforms of APX, with peroxisomal and cytosolic APX being indistinguishable from one another on this blot. The three bands in each blot from the top to bottom correspond to thylakoid (t), stromal (s), and both peroxisomal and cytosolic (p+c) APX. When tAPX and sAPX bands came up weak during a short exposure time (g-h), the same membrane was exposed for a longer time (i-j). The quantitative analysis of immunoblot signals from both light shifts is summarized in Supplementary Data 2-3.

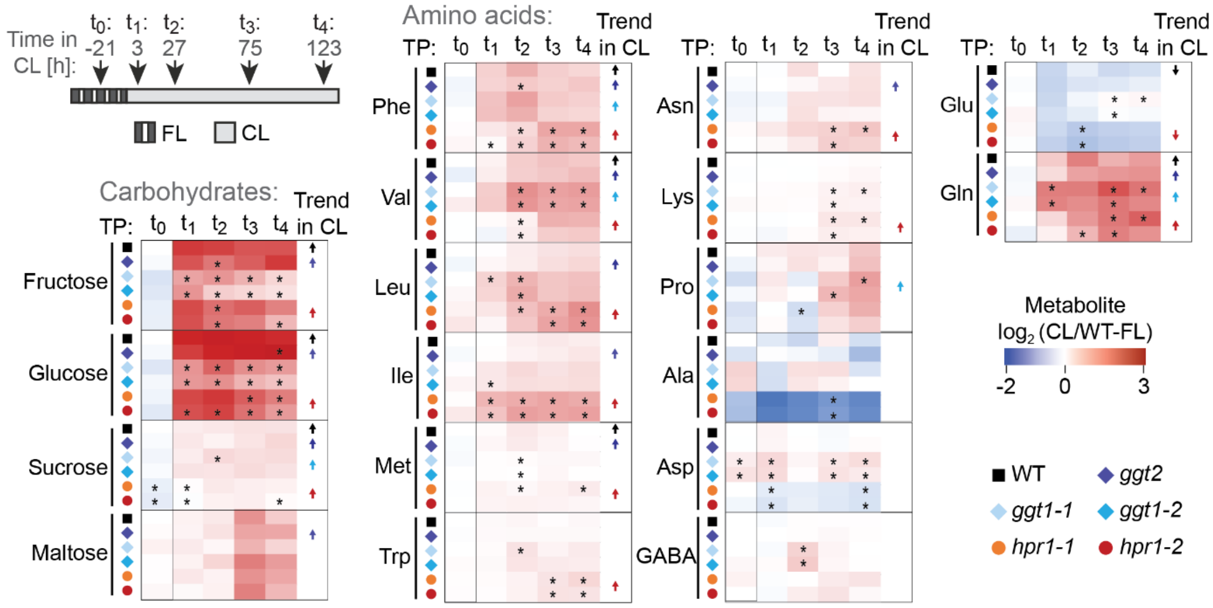

Supplementary Figure 11. Changes in metabolite levels in response to a shift from fluctuating to control light.

WT, *ggt2* and two mutant alleles of each *ggt1* and *hpr1* were grown under fluctuating light (FL: 1 min 700 µmol photons m^{-2} s^{-1}, 4 min 70 µmol photons m^{-2} s^{-1}) for 35 d and then shifted to control light (CL: 200 µmol photons m^{-2} s^{-1}) on day 36. Samples for metabolite extraction were taken 6 h into the light period at 21 h before (t_0) and 3 h (t_1), 27 h (t_2), 75 h (t_3) and 123 h (t_4) after the light shift. Heatmaps of log_2 transformed metabolite averages normalized on WT in FL are shown for carbohydrates and free amino acids. Averages of n = 3-4 are shown. Exact number of replicates per group can be found in Supplementary Data 4. Asterisks indicate significant differences between all analyzed mutant alleles and WT at the given time point. Upwards pointing arrows in the corresponding color (WT: black; *ggt2*: dark blue; both *ggt1* lines: light blue; both *hpr1* lines: red) indicate significant differences to t_0 of the same genotype for at least two subsequent time points in CL as determined via two-way ANOVA and post-hoc Tukey multiple comparison test with $p < 0.05$. The complete data set can be found in Supplementary Data 4. Ala – Alanine, Asn – Asparagine, Asp – Aspartic acid, GABA – Y-aminobutyric acid, Glu – Glutamic acid, Gln – Glutamine, Ile – Isoleucine, Leu – Leucine, Lys – Lysine, Met – Methionine, Phe – Phenylalanine, Pro – Proline, Trp – Tryptophan, Val – Valine, 2-OG – 2-oxoglutarate, TP – Time point.

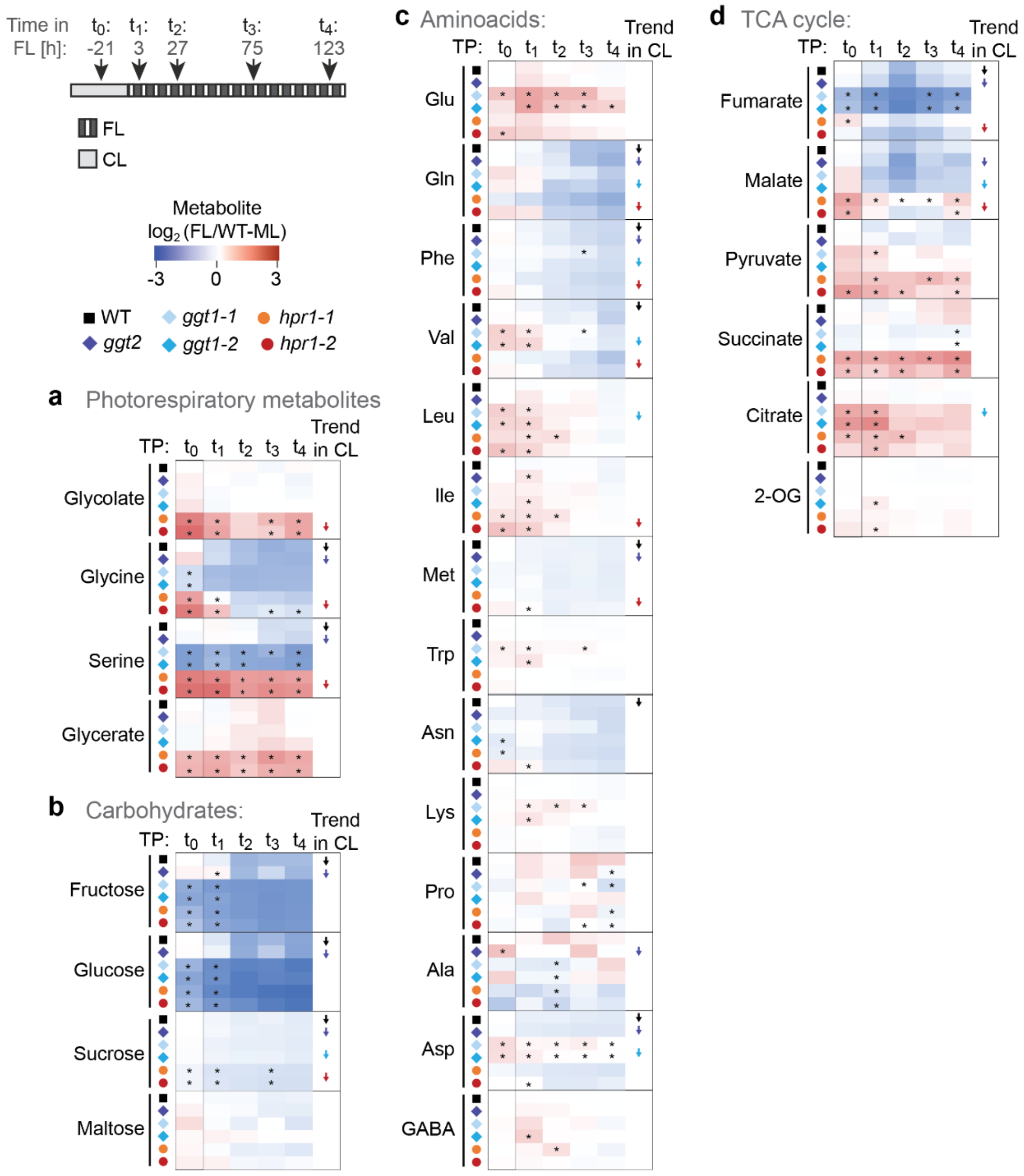

Supplementary Figure 12. Changes in metabolite levels in response to a shift from control to fluctuating light.

a-d. WT, *ggt2* and two mutant alleles of each *ggt1* and *hpr1* were grown under control light (CL: 200 µmol photons m^{-2} s^{-1}) for 29 d and then shifted to fluctuating light (FL: 1 min 700 µmol photons m^{-2} s^{-1}, 4 min 70 µmol photons m^{-2} s^{-1}) on day 30. Samples for metabolite extraction were taken 6 h into the light period at 21 h (t_0) before and 3 h (t_1), 27 h (t_2), 75 h (t_3) and 123 h (t_4) after the light shift. Heatmaps of $\log_2$ transformed metabolite averages (n = 3-4) normalized on WT in CL are shown for photorespiratory metabolites (a), carbohydrates (b), amino acids (c) and TCA cycle metabolites (d). Averages of n = 3-4 are shown. Exact number of replicates per group can be found in Supplementary Data 4. Asterisks indicate significant differences between all analyzed mutant alleles and WT at the given time point. Downwards pointing arrows in the corresponding color (WT: black; *ggt2*: dark blue; both *ggt1* lines: light blue; both *hpr1* lines: red) indicate significant differences to t_0 of the same genotype for at least two subsequent time points in FL as determined via two-way ANOVA and post-hoc Tukey multiple comparison tests with $p < 0.05$. The complete data set can be found in Supplementary Data 4. Ala – Alanine, Asn – Asparagine, Asp – Aspartic acid, GABA – Y-aminobutyric acid, Glu – Glutamic acid, Gln – Glutamine, Ile – Isoleucine, Leu – Leucine, Lys – Lysine, Met – Methionine, Phe – Phenylalanine, Pro – Proline, Trp – Tryptophan, Val – Valine, 2-OG – 2-oxoglutarate, TP – Time point.

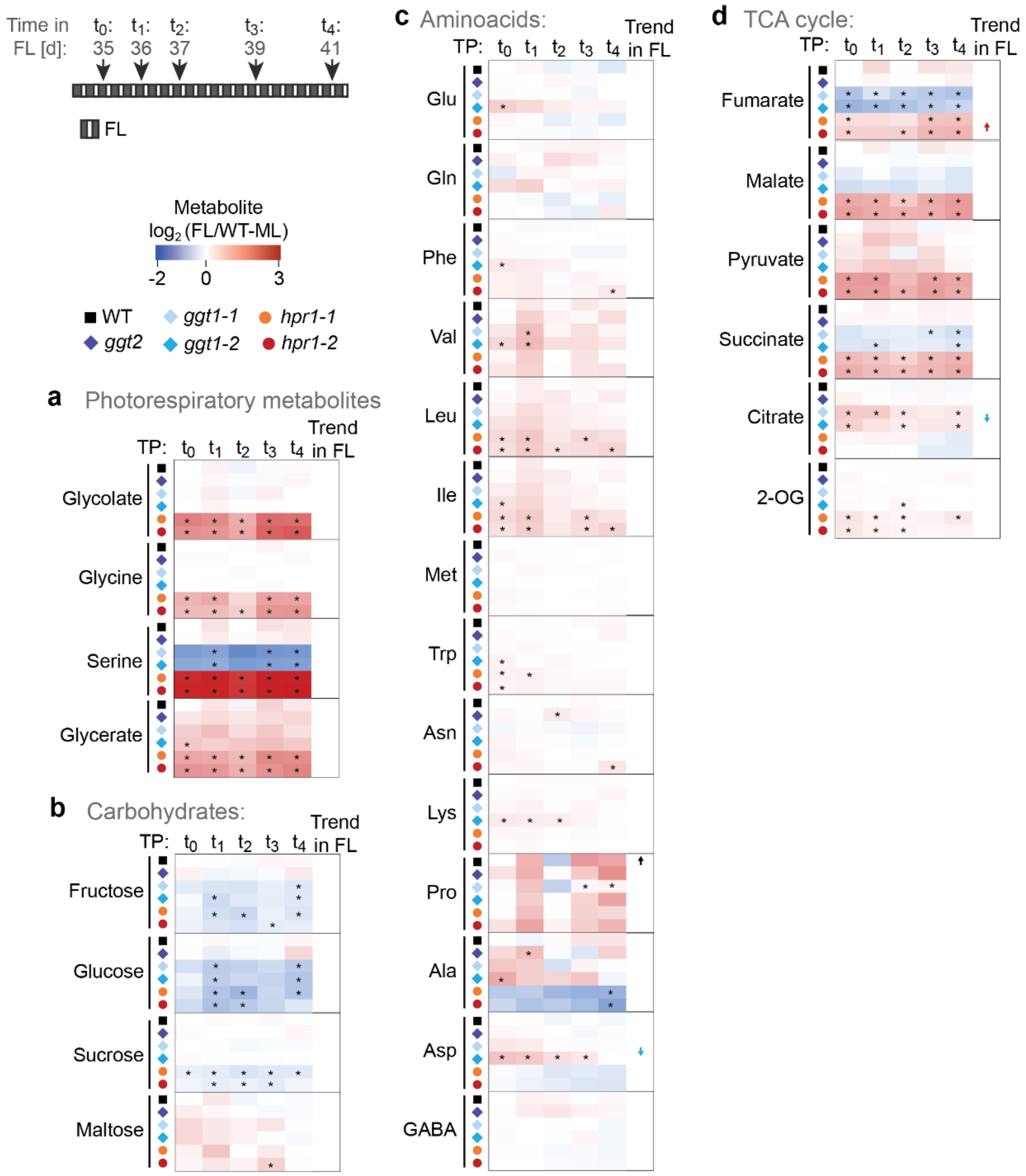

Supplementary Figure 13. Relative metabolite levels at comparable sampling time points of non-shifted, fluctuating light-grown plants.

a-d. WT, *ggt2* and two mutant alleles of each *ggt1* and *hpr1* were grown under fluctuating light (FL: 1 min 700 µmol photons m^{-2} s^{-1}, 4 min 70 µmol photons m^{-2} s^{-1}) only. Samples for metabolite extraction were taken at the same time points as in Fig. 4. Heatmaps of log_2 transformed metabolite averages normalized on WT at t_0 are shown for photorespiratory metabolites (a), carbohydrates (b), amino acids (c) and TCA cycle metabolites (d). Averages of n = 3-4 are shown. Exact number of replicates per group can be found in Supplementary Data 4. Asterisks indicate significant differences between all analyzed mutant alleles and WT at the given time point. Upwards or downwards pointing arrows in the corresponding color (WT: black; *ggt2*: dark blue; both *ggt1* lines: light blue; both *hpr1* lines: red) indicate significant differences to t_0 of the same genotype for at least two subsequent time points in t_1-t_4 as determined via two-way ANOVA and post-hoc Tukey multiple comparison tests with $p < 0.05$. The complete data set can be found in Supplementary Data 4. Ala – Alanine, Asn – Asparagine, Asp – Aspartic acid, GABA – Y-aminobutyric acid, Glu – Glutamic acid, Gln – Glutamine, Ile – Isoleucine, Leu – Leucine, Lys – Lysine, Met – Methionine, Phe – Phenylalanine, Pro – Proline, Trp – Tryptophan, Val – Valine, 2-OG – 2-oxoglutarate, TP – Time point.

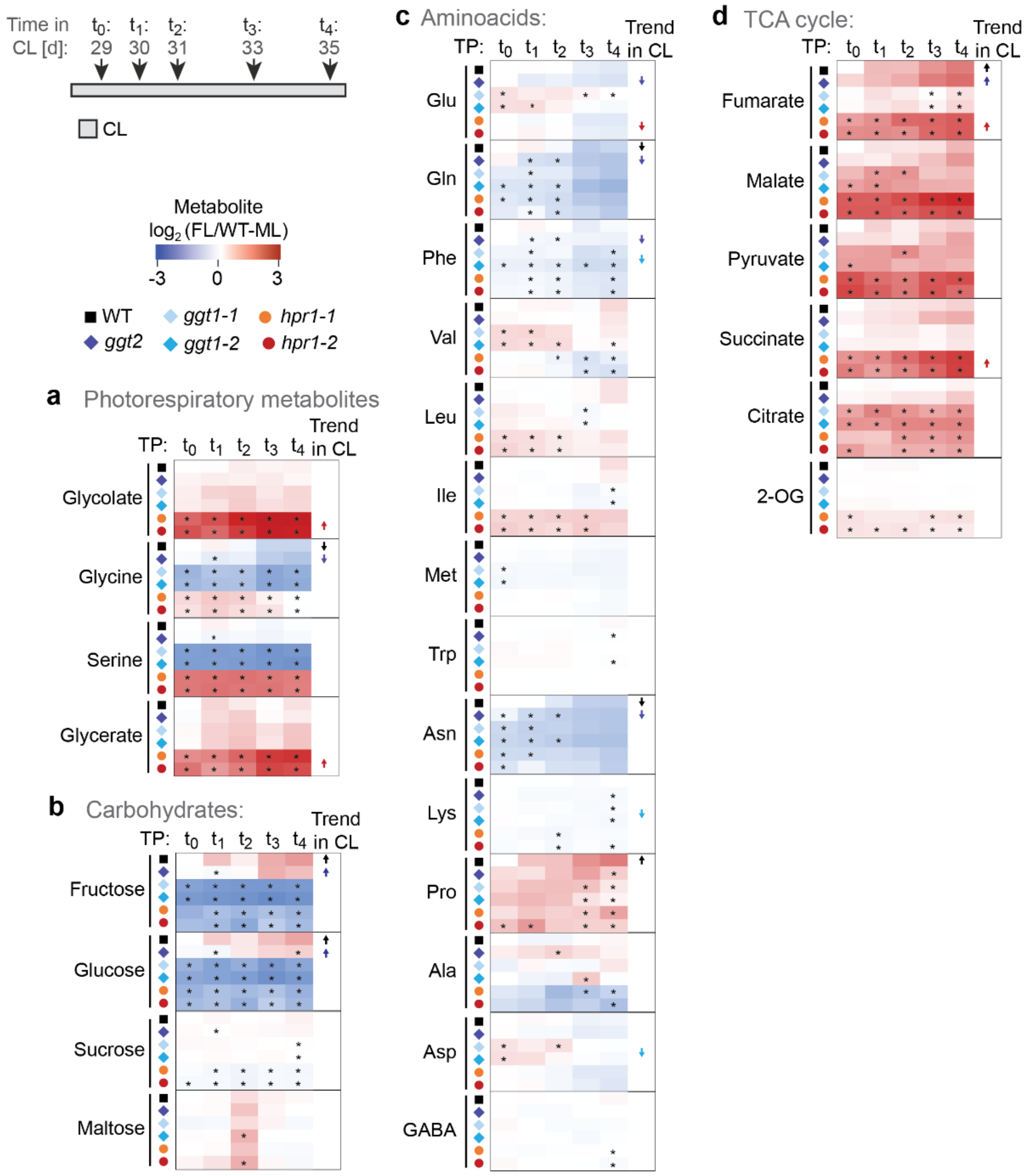

Supplementary Figure 14. Relative metabolite levels at comparable sampling time points of non-shifted, control light-grown plants.

a-d. WT, *ggt2* and two mutant alleles of each *ggt1* and *hpr1* were grown under control light (CL: 200 µmol photons m^{-2} s^{-1}) only. Samples for metabolite extraction were taken at the same time points as in Supplementary Fig. 12. Heatmaps of log$_2$ transformed metabolite averages normalized on WT at t_0 are shown for photorespiratory metabolites (a), carbohydrates (b), amino acids (c) and TCA cycle metabolites (d). Averages of n = 3-4 are shown. Exact number of replicates per group can be found in Supplementary Data 4. Asterisks indicate significant differences between all analyzed mutant alleles and WT at the given time point. Upwards or downwards pointing arrows in the corresponding color (WT: black; *ggt2*: dark blue; both *ggt1* lines: light blue; both *hpr1* lines: red) indicate significant differences to t_0 of the same genotype for at least two subsequent time points in t_1-t_4 as determined via two-way ANOVA and post-hoc Tukey multiple comparison tests with $p < 0.05$. The complete data set can be found in Supplementary Data 4. Ala – Alanine, Asn – Asparagine, Asp – Aspartic acid, GABA – Y-aminobutyric acid, Glu – Glutamic acid, Gln – Glutamine, Ile – Isoleucine, Leu – Leucine, Lys – Lysine, Met – Methionine, Phe – Phenylalanine, Pro – Proline, Trp – Tryptophan, Val – Valine, 2-OG – 2-oxoglutarate, TP – Time point.

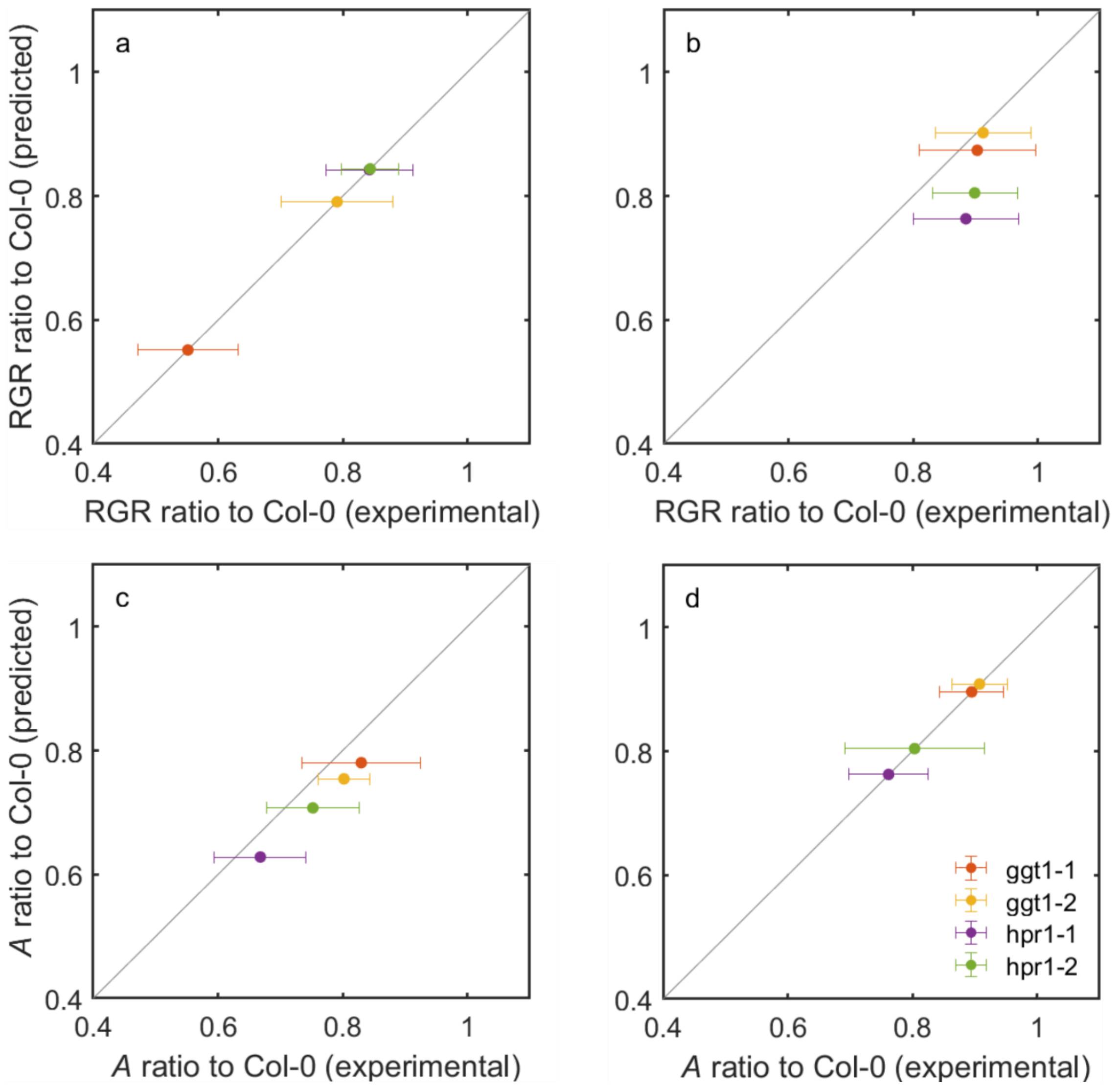

Supplementary Figure 15. Correspondence between experimental and predicted relative growth rates and net CO_2 assimilation rates.

a-b. Predicted and experimentally determined ratios in relative growth rate (RGR) between the four mutants and the WT for constant light (CL: 200 µmol photons m^{-2} s^{-1}; a) and fluctuating light (FL: 1 min 700 µmol photons m^{-2} s^{-1}, 4 min 70 µmol photons m^{-2} s^{-1}; b). The predicted ratio in RGR between the FL and CL conditions for the WT was 0.70 while the experimental ratio was 0.74. The error bars represent the standard deviation across ten samples per genotype. **c-d.** Similar to panels a-b, the experimentally determined and predicted net CO_2 assimilation rates (*A*) were divided by the WT value in CL (c) and FL (d) condition. The Spearman correlation between the unscaled experimental and predicted values was 1.0 (p = 0.0167) for both conditions. The predicted ratio in *A* between the FL and CL conditions for the WT was 0.46 while the experimental ratio was 0.48. The error bars represent the standard deviation across n = 4 (CL: *hpr1-2*; FL: *ggt1-1*, *hpr1-2*), n = 5 (CL: *ggt1-1*; FL: *hpr1-1*) or n = 6 (CL: *ggt1-2*, *hpr1-1*; FL: *ggt1-2*) samples.

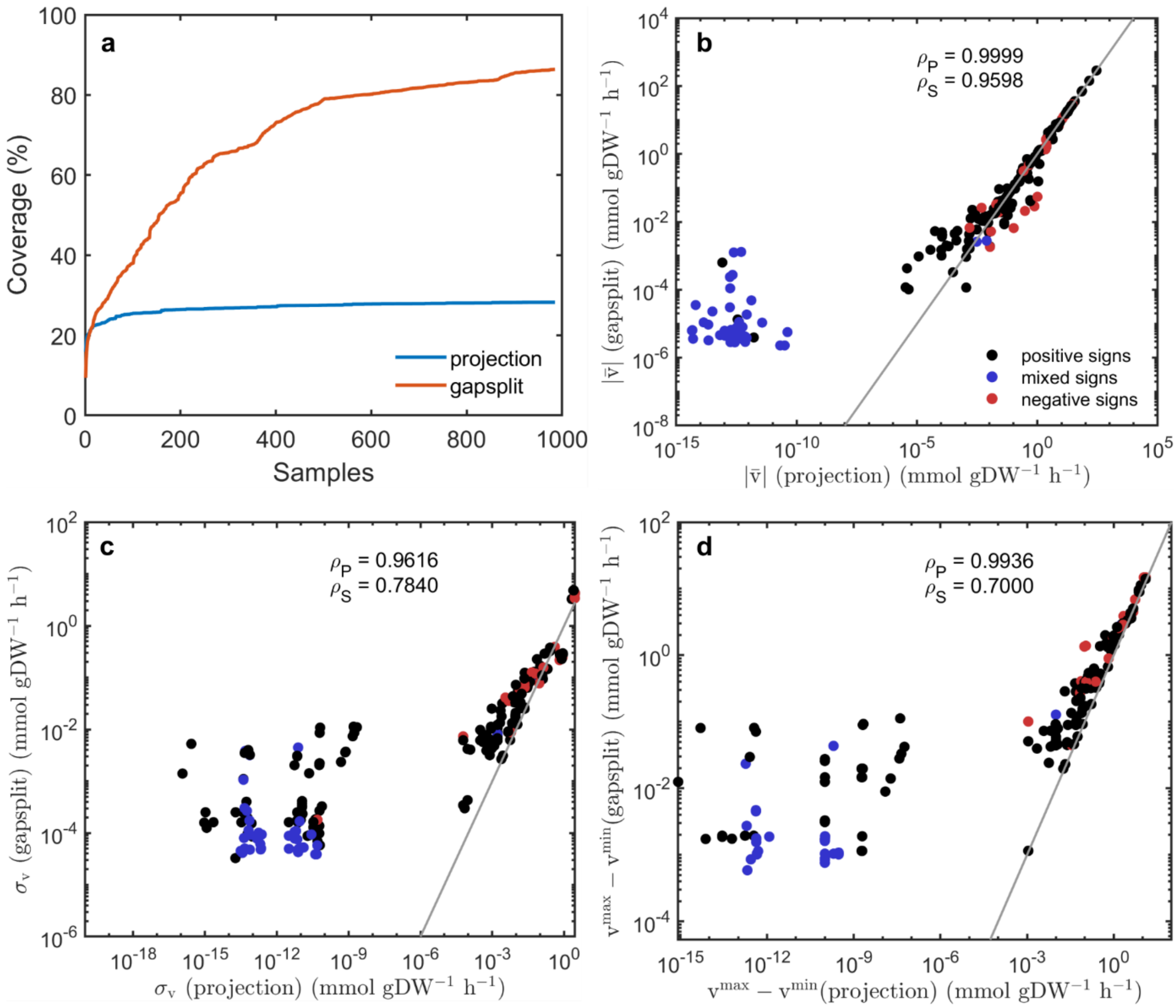

Supplementary Figure 16. Comparison of flux sampling results with gapsplit for one representative optimization problem.

The first optimization problem (i.e., Col-0, CL) was used as input for the gapsplit sampler[3] with default settings. The same variables for which random vectors were projected onto the feasible space were used as primary targets in gapsplit (i.e., net flux variables with $v^{max} - v^{min} \geq 10^{-5}\ mmol\ gDW^{-1}h^{-1}$, n = 502). **a.** Comparison of the "projection" sampling approach used in this study and the gapsplit sampler sampling by coverage defined by 1 – average relative maximum gap (considering only the net flux variables, number of samples: 985). **b-d.** Absolute average fluxes (b), standard deviations (c), and ranges (differences between minimum and maximum flux; d) over all samples are shown. Black and red color indicate agreement in positive and negative sign, respectively (81.7% and 6.8%, total 88.4%), and blue color indicates disagreement in signs (11.6%). The gray lines depict perfect agreement.

2. Supplementary methods

Detailed workflow to model flux distributions in photorespiratory mutants

The flux distributions were predicted following the workflow outlined below:

1. **Optimize v_{bio} for wild type model (under control light)**

The TMFA problem is solved to obtain a thermodynamically feasible flux distribution that yields the highest predicted relative growth rate. The optimization problem contains additional constraints on the ratio between the Rubisco oxygenation and carboxylation reactions (fixed within one standard deviation, $\sigma_{\phi}^{CL} = 0.087$, $\sigma_{\phi}^{FL} = 0.059$) and photon uptake (Supplementary Data 8). The upper limit for the photon uptake was determined by scaling the reaction upper bound of 1000 mmol gDW^{-1} h^{-1} by the ratio of the experimental light intensities (CL: 200 µmol photons m^{-2} s^{-1}, FL: 90 µmol photons m^{-2} s^{-1}) by a saturating light intensity of 700 µmol photons m^{-2} s^{-1}.

$$\max v_{bio}$$

s.t.

$$\mathbf{Sv} = \mathbf{0} \tag{1}$$

$$\mathbf{v^{min}} \leq \mathbf{v} \leq \mathbf{v^{max}} \tag{2}$$

$$\mathbf{v} - K\mathbf{y} < \mathbf{0} \tag{3}$$

$$\mathbf{\Delta_r G} - K + K\mathbf{y} < \mathbf{0} \tag{4}$$

$$\mathbf{\Delta_r G} = \mathbf{\Delta_r G^\circ} + \mathbf{S^T \mu} \tag{5}$$

$$\mathbf{x^{min}} \leq \mathbf{x} \leq \mathbf{x^{max}} \tag{6}$$

$$\mathbf{y} \in \{0,1\} \tag{7}$$

$$\phi - 1.1 \cdot \sigma_{\phi} \leq \frac{v_o}{v_c} \leq \phi + 1.1 \cdot \sigma_{\phi} \tag{8}$$

$$v_{hv} \leq v_{hv}^{max}.$$

The optimal value, i.e., maximum relative growth rate, is denoted by v_{bio}^{opt}.

The constraints in Eq. (8) define the lower and upper limits for the ratio between the Rubisco oxygenation and carboxylation reaction, respectively. The values for ϕ and σ_{ϕ} were specific for the control light condition. A tolerance of 10% was added to ensure feasibility of this and the following optimization problems.

2. **Set lower bound for relative growth rate**
3. **Set concentration limits for measured metabolites**

Measured metabolite concentrations as converted from metabolite abundances used to limit metabolite concentrations in the TMFA program within one standard deviation:

$$x^{min} = x - \sigma_x$$

$$x^{max} = x + \sigma_x.$$

4. Check if program is feasible

Yes → proceed to step 5

No → relax constraints on metabolite concentrations by solving the following program:

$$\max v_{bio} - 0.001 \sum_{i \in M(measured)} |\varepsilon_i|$$

s.t.

Eq. (1)-(7) (TMFA constraints)

$$x_i^{min} - \varepsilon_i \leq x_i \leq x_i^{max} + \varepsilon_i, \forall i \in M(measured)$$

$$\phi - 1.1 \cdot \sigma_\phi \leq \frac{v_o}{v_c} \leq \phi + 1.1 \cdot \sigma_\phi$$

$$v_{hv} \leq v_{hv}^{max}$$

$$v_{bio} \geq (1 - 0.001) \cdot v_{bio}^{opt}.$$

The program above introduces relaxations (ε) to the concentration limits of measured metabolites and maximizes the weighted sum of relative growth rate and the sum of relaxations.

The constraint $v_{bio} \geq (1 - 0.001) \cdot v_{bio}^{opt}$ imposes a lower bound on the flux through the biomass reaction which is 99.9% of the optimal value determined in step 1. The tolerance is introduced here and in the following steps (where indicated) to ensure feasibility of the respective optimization problem.

Once $\boldsymbol{\varepsilon}$ has been determined, the metabolite concentrations are re-calculated and the lower and upper limits for metabolite concentrations updated as follows:

$$x_i^{min'} = 0.9 \cdot \left(x_i^{min} - \varepsilon_i\right), \forall i \in M(measured)$$

$$x_i^{max'} = 1.1 \cdot (x_i^{max} + \varepsilon_i), \forall i \in M(measured).$$

5. Solve TMFA problem, from step 1, above with (relaxed) measured metabolite concentrations

After solving the problem, v_{bio}^{opt} is updated.

6. Minimize the sum of fluxes in the wild type model

$$\min z = \sum_{j \in R \setminus v_{bio}} |v_j|$$

s.t.

Eq. (1)-(7) (TMFA constraints)

$$x_i^{min'} \leq x_i \leq x_i^{max'}, \forall i \in M(measured)$$

$$\phi - 1.1 \cdot \sigma_\phi \leq \frac{v_o}{v_c} \leq \phi + 1.1 \cdot \sigma_\phi$$

$$v_{hv} \leq v_{hv}^{max}$$

$$v_{bio} \geq (1 - 0.001) \cdot v_{bio}^{opt}.$$

The optimal value, i.e. minimum distance is denoted z^{opt}.

7. **Set upper bound for the sum of fluxes**
8. **Determine feasible flux ranges using TVA**

   For each reaction j the following problems are solved:

$$\min/\max v_j$$

s.t.

Eq. (1)-(7) (TMFA constraints)

$$x_i^{min'} \leq x_i \leq x_i^{max'}, \forall i \in M(measured)$$

$$\phi - 1.1 \cdot \sigma_\phi \leq \frac{v_o}{v_c} \leq \phi + 1.1 \cdot \sigma_\phi$$

$$v_{hv} \leq v_{hv}^{max}$$

$$v_{bio} \geq (1 - 0.001) \cdot v_{bio}^{opt}$$

$$z \leq (1 + 0.003) \cdot z^{opt}.$$

The constraint $z \leq (1 + 0.003) \cdot z^{opt}$ imposes an upper limit on the value of z, which is 100.3% of the previously determined optimum z^{opt} (step 6) to ensure feasibility of the TVA programs.

9. **Sample random flux distributions that satisfy the constraints from step 7**

   Generate a vector v^* containing random fluxes for each reaction within the feasible ranges determined in step 7. Subject to all constraints from step 7, a minimization problem is solved to find a flux distribution that minimizes the distance to v^* ($\min|v^* - v|$). This problem is solved with 1000 different v^* samples to obtain 1000 feasible flux distributions.
10. **Model flux distributions of photorespiratory mutants**
    a. **Block reactions associated with knockout by setting lower and upper flux bound to zero**
    b. **Fix v_{bio} according to ratio to the relative growth rate of Col-0 wild type**

The optimal growth rate determined for the wild type in step 5 (v_{bio}^{opt}) is referred to as v_{bio}^{wt}. The relative growth rate of the mutant is fixed between a lower and an upper bound, which are calculated as follows:

$$v_{bio}^{mut,min} = (1 - 10^{-3}) \cdot v_{bio}^{wt} \cdot \frac{RGR^{mut}}{RGR^{wt}}$$

and

$$v_{bio}^{mut,max} = (1 + 10^{-3}) \cdot v_{bio}^{wt} \cdot \frac{RGR^{mut}}{RGR^{wt}}.$$

c. **Fix net CO_2 assimilation rate according to ratio of A to Col-0 wild type**

Similar to the relative growth rate, the ratio on A is fixed using the measured values for the wild type and the respective mutant. The net CO_2 assimilation rate predicted by the model is approximated by $v_c - 0.5v_o - v_{CO_2(m \to c)}$, where v_c and v_o are the fluxes through the Rubisco carboxylation and oxygenation reactions, and $v_{CO_2(m \to c)}^{mut}$ export flux of CO_2 from the mitochondrion to the cytosol, which approximates dark respiration[4]. The lower and upper bounds on net CO_2 assimilation rate are given by:

$$A^{mut,min} = (1 - 10^{-3}) \cdot \left(v_c^{wt} - 0.5v_o^{wt} - v_{CO_2(m \to c)}^{wt}\right) \cdot \frac{A^{mut}}{A^{wt}},$$

$$A^{mut,max} = (1 + 10^{-3}) \cdot \left(v_c^{wt} - 0.5v_o^{wt} - v_{CO_2(m \to c)}^{wt}\right) \cdot \frac{A^{mut}}{A^{wt}}.$$

Fluxes with superscript "wt" originate from the flux distribution predicted in step 5, while values for A were determined experimentally (Supplementary Data 8).

d. **Add constraints on oxygenation to carboxylation ratio and photon uptake**

oxygenation to carboxylation ratio:

$$\phi - 1.1 \cdot \sigma_\phi \le \frac{v_o}{v_c} \le \phi + 1.1 \cdot \sigma_\phi$$

photon uptake

$$v_{hv} \le v_{hv}^{max}$$

e. **Solve the resulting TMFA problem**

$$\max v_{bio}$$

s.t.

Eq. (1)-(7) (TMFA constraints)

$$\phi - 1.1 \cdot \sigma_\phi \le \frac{v_o}{v_c} \le \phi + 1.1 \cdot \sigma_\phi$$

$$v_{hv} \le v_{hv}^{max}$$

$$v_{bio}^{mut,min} \le v_{bio} \le v_{bio}^{mut,max}$$

$$A^{mut,min} \le v_c - 0.5v_o - v_{CO_2(m \to c)} \le A^{mut,max}$$

f. **Set concentration limits for measured metabolites (see step 2)**

g. **Check if program is feasible**

Yes → proceed to step 10i

No → relax constraints on metabolite concentrations (see step 4, above)

h. **Check if program is feasible**

Yes → proceed to step 10i

No → relax constraints on v_{bio}

The following program minimizes the absolute difference between the expected growth rate v_{bio}^{exp}

$$v_{bio}^{exp} = v_{bio}^{wt} \frac{RGR^{mut}}{RGR^{wt}}$$

and the sum of metabolite concentration relaxation as a weighted sum:

$$\min\left|v_{bio}^{exp} - v_{bio}\right| + 0.01 \sum_{i \in M(measured)} |\varepsilon_i|$$

s.t.

Eq. (1)-(7) (TMFA constraints)

$$\phi - 1.1 \cdot \sigma_\phi \le \frac{v_o}{v_c} \le \phi + 1.1 \cdot \sigma_\phi$$

$$v_{hv} \le v_{hv}^{max}$$

$$A^{mut,min} \le v_c - 0.5 v_o - v_{CO_2(m \to c)} \le A^{mut,max}$$

$$x_i^{min} - \varepsilon_i \le x_i \le x_i^{max} + \varepsilon_i, \forall i \in M(measured).$$

The determined value for v_{bio} is then used to update the lower and upper bounds on the predicted relative growth rate of the mutant:

$$v_{bio}^{mut,min} = (1 - 10^{-3}) \cdot v_{bio}$$

$$v_{bio}^{mut,max} = (1 + 10^{-3}) \cdot v_{bio}.$$

i. **Solve TMFA problem again with (relaxed) measured metabolite concentrations (see step 5) and**

j. **Minimize the distance to the wild type flux distribution**

The following optimization problem finds the flux distribution with the smallest distance to the wild type flux distribution obtained in step 6:

$$\min z = \sum_{j \in R \setminus v_{bio}} \left|v_j^{mut} - v_j^{wt}\right|$$

s.t.

Eq. (1)-(7) (TMFA constraints)

$$x_i^{min'} \leq x_i \leq x_i^{max'}, \forall i \in M(measured)$$

$$\phi - 1.1 \cdot \sigma_\phi \leq \frac{v_o}{v_c} \leq \phi + 1.1 \cdot \sigma_\phi$$

$$v_{hv} \leq v_{hv}^{max}$$

$$v_{bio}^{mut,min} \leq v_{bio} \leq v_{bio}^{mut,max}$$

$$A^{mut,min} \leq v_c - 0.5 v_o - v_{CO_2(m \to c)} \leq A^{mut,max}.$$

The minimum distance obtained from the solution to the problem above is called z^{opt}.

k. Set the upper bound for the distance to the wild type flux distribution, obtained in step 10j

l. Determine feasible flux ranges using TVA

For each reaction j the following problems are solved:

$$\min/\max v_j$$

s.t.

Eq. (1)-(7) (TMFA constraints)

$$x_i^{min'} \leq x_i \leq x_i^{max'}, \forall i \in M(measured)$$

$$\phi - 1.1 \cdot \sigma_\phi \leq \frac{v_o}{v_c} \leq \phi + 1.1 \cdot \sigma_\phi$$

$$v_{hv} \leq v_{hv}^{max}$$

$$v_{bio}^{mut,min} \leq v_{bio} \leq v_{bio}^{mut,max}$$

$$A^{mut,min} \leq v_c - 0.5 v_o - v_{CO_2(m \to c)} \leq A^{mut,max}$$

$$z \leq (1 + 0.002) \cdot z^{opt}$$

m. Sample random flux distributions that satisfy the constraints from step 10l

See step 9 for objective and explanation.

11. Create TMFA problem for FL-specific wild type model

The values for ϕ and v_{hv}^{max} for the Col-0 wild type in FL were used as constraints as in step 1.

12. Fix v_{bio} according to ratio of RGR to Col-0 wild type in CL

The optimal growth rate determined for the wild type in CL (step 5, v_{bio}^{opt}) is referred to as v_{bio}^{CL}. The relative growth rate of the wild type in FL is fixed between a lower and an upper bound, which are calculated by:

$$v_{bio}^{FL,min} = (1 - 10^{-3}) \cdot v_{bio}^{CL} \cdot \frac{RGR^{wt,Fl}}{RGR^{wt,CL}}$$

and

$$v_{bio}^{FL,max} = (1 + 10^{-3}) \cdot v_{bio}^{CL} \cdot \frac{RGR^{wt,FL}}{RGR^{wt,CL}}.$$

13. Fix net CO_2 assimilation rate according to ratio of A to Col-0 wild type in CL

Similar to the relative growth rate, the ratio on A is fixed using the measured values for the wild type in CL and FL (see step 10c). The lower and upper bounds on net CO_2 assimilation rate are given by:

$$A^{FL,min} = (1 - 10^{-3}) \cdot \left(v_c^{CL} - 0.5v_o^{CL} - v_{CO_2(m\to c)}^{CL}\right) \cdot \frac{A^{wt,FL}}{A^{wt,CL}},$$

$$A^{FL,max} = (1 + 10^{-3}) \cdot \left(v_c^{CL} - 0.5v_o^{CL} - v_{CO_2(m\to c)}^{CL}\right) \cdot \frac{A^{wt,FL}}{A^{wt,CL}}.$$

Fluxes with superscript “CL” originate from the flux distribution predicted in step 5, while values for A were determined experimentally (Supplementary Data 8).

14. Check if program is feasible

Yes → Proceed to step 15

No → relax constraint on relative growth rate

The following optimization program finds a flux distribution that yields the relative growth rate that is closest to the expected relative growth rate v_{bio}^{exp}:

$$v_{bio}^{exp} = v_{bio}^{wt,CL} \frac{RGR^{wt,FL}}{RGR^{wt,CL}}.$$

The upper bound on the photon uptake reaction is alleviated to allow for an increase in predicted relative growth rate:

$$\min\left|v_{bio}^{exp} - v_{bio}\right|$$

s.t.

Eq. (1)-(7) (TMFA constraints)

$$\phi - 1.1 \cdot \sigma_\phi \leq \frac{v_o}{v_c} \leq \phi + 1.1 \cdot \sigma_\phi$$

$$A^{FL,min} \leq v_c - 0.5v_o - v_{CO_2(m\to c)} \leq A^{FL,max}$$

The obtained value for v_{bio} is called v_{bio}^{opt}.

Next, the flux through v_{hv} is minimized subject to the constraints from the program shown above, while keeping v_{bio} at v_{bio}^{opt} (± 0.1%). The updated value for v_{hv} is used for all subsequent simulations for FL.

15. Repeat steps 3-10 for the FL condition with constraints on v_{bio} and A

Effects of alternative flux sampling

In the paper that accompanies the matTFA toolbox[5], which we used to construct the TMFA model, the authors fixed the directions of bidirectional reactions to be able to use the artificial centering hit-and-run (ACHR) sampler implemented as part of the COBRA toolbox. Since this procedure decreases the feasible space to be sampled in a biased way, we developed the sampling procedure described in step 9 above, to directly use the TMFA problem for sampling. While this approach allows a fast flux sampling, we are aware that it may guarantee uniform sampling of the feasible space.

For a comparison, we applied the gapsplit sampler[3], which can deal with mixed-integer linear problems. One representative optimization problem (Col-0, CL) was exported and used as input for the gapsplit sampler with default settings. All net flux variables (the same variables, for which random vectors we projected onto the solution space) were selected as primary targets whose distributions in the feasible space are explored by gapsplit. To ensure feasibility of the second norm minimization for the secondary targets, we first identified and corrected an error in the gapsplit code and set an upper limit for the minimization weights of 10,000. The performances of the two sampling strategies, gapsplit and our projection-based method, were compared by using the coverage, defined as follows[3,6]:

$$\text{coverage} = 1 - \text{mean}(\text{relative max gap}(x_i))\,.$$

The term $\max\text{gap}(x_i)$ denotes the maximum difference between any two sampled values including the limits of the feasible ranges of a variable x_i. The relative max gap is then calculated by scaling $\max\text{gap}(x_i)$ by the feasible range of x_i ($\max(x_i) - \min(x_i)$).

We observed that our sampling strategy ("projection"), described in step 9, only reached a maximum coverage of 28.25% with 985 samples, while, with the same number of samples, the gapsplit sampler reached a coverage of 86.4% (Supplementary Fig. 16). We further note that the "projection" sampling uses first norm minimization to minimize the distance between the random flux vector and the feasible space, while the gapsplit sampler uses second norm minimization to minimize the distance of average gaps of secondary targets to the feasible space (with fixed primary target). The use of the second norm results in many small differences, while using the first norm results in fewer, large differences. Hence, the use of the second norm in gapsplit increases the coverage by adding many small differences, but these may not necessarily affect the average flux values that we compared in this study (in different modeling scenarios).

To investigate whether or not the difference in sampling coverage yields significant changes with respect to average fluxes, we compared the average values of all net flux variables (Supplementary Fig. 16b). We found that the average flux values obtained from the two sampling approaches correlated perfectly, with a Pearson correlation of $\rho = 0.9999$. Notably, some fluxes only showed very low average flux values ($< 10^{-10}\, mmol\ gDW^{-1}\ h^{-1}$) when using the "projection" sampling, which is a result of incomplete coverage, already discussed above. These reactions are also mainly responsible for the disagreement in sign of the respective averages in flux values between the two approaches, which otherwise show high agreement (88.4%) of the flux averages across the reactions in the model. Further, the standard deviations (Supplementary Fig. 16c) and ranges (difference between maximum and

minimum sampled flux; Supplementary Fig. 16d) agree with high Pearson correlations of 0.96 and 0.99, respectively. From Supplementary Fig. 16c-d, it is then obvious that the disagreements in sign result from the very narrow ranges (and small standard deviations) for some of the reactions, which cause numerical fluctuations around average fluxes that are essentially equal to zero – that can be readily neglected.

Five reactions with average fluxes below $10^{-10}\,mmol\,gDW^{-1}\,h^{-1}$ from the projection sampling are presented in Figure 5; however, the square root of the average flux obtained using gapsplit does not exceed $0.04\,mmol\,gDW^{-1}\,h^{-1}$, which is very low compared to the remaining fluxes that are shown. These include the following: The chloroplast HPR3 reaction showed $\sqrt{v} = 0.04\,mmol\,gDW^{-1}\,h^{-1}$, which is in line with the other very low-magnitude fluxes shown in the heatmap. Three reactions are involved in the conversion of 2-oxoglutarate to succinate and had $\sqrt{v} = 0.005\,mmol\,gDW^{-1}\,h^{-1}$. The fifth reaction is the mitochondrial NADH-dependent isocitrate dehydrogenase, which had $\sqrt{v} = 0.0026\,mmol\,gDW^{-1}\,h^{-1}$. While these low average fluxes would have caused dark blue coloring in the heatmaps in Figure 5, they would still have been considered marginally active.

From these results, we conclude that while the use of a different sampling strategy (i.e., gapsplit) provided a better coverage of the feasible flux space, the used projection sampling approach does not affect the presented findings with respect to the average fluxes as well as standard deviations per reaction.

3. Supplementary References

1 Obayashi, T., Hibara, H., Kagaya, Y., Aoki, Y. & Kinoshita, K. ATTED-II v11: A Plant Gene Coexpression Database Using a Sample Balancing Technique by Subagging of Principal Components. *Plant & cell physiology* **63**, 869-881 (2022).

2 Winter, D. *et al.* An “Electronic Fluorescent Pictograph” Browser for Exploring and Analyzing Large-Scale Biological Data Sets. *PLOS ONE* **2**, e718 (2007).

3 Keaty, T. C. & Jensen, P. A. Gapsplit: efficient random sampling for non-convex constraint-based models. *Bioinformatics (Oxford, England)* **36**, 2623-2625 (2020).

4 Farquhar, G. D., von Caemmerer, S. & Berry, J. A. A biochemical model of photosynthetic CO2 assimilation in leaves of C3 species. *Planta* **149**, 78-90 (1980).

5 Salvy, P. *et al.* pyTFA and matTFA: a Python package and a Matlab toolbox for Thermodynamics-based Flux Analysis. *Bioinformatics (Oxford, England)* **35**, 167-169 (2018).

6 Binns, M., de Atauri, P., Vlysidis, A., Cascante, M. & Theodoropoulos, C. Sampling with poling-based flux balance analysis: optimal versus sub-optimal flux space analysis of Actinobacillus succinogenes. *BMC Bioinformatics* **16**, 49 (2015).
