# Supplementary material for: Growth in fluctuating light buffers plants against photorespiratory perturbations
Source: Nat Commun. 2023 Nov 3;14:7052. doi: 10.1038/s41467-023-42648-x (PMC10624928; doi:10.1038/s41467-023-42648-x)
Supplement: Supplementary file 3 — Description of Additional Supplementary Files [file 41467_2023_42648_MOESM3_ESM.pdf]

Description of Additional Supplementary Files

Supplementary Data 1: Statistical analysis of gas exchange parameters of LL, FL and CL grown plants.
Gas exchange measurements were performed on WT, *ggt2* and two mutant alleles each of *ggt1* and *hpr1*. All plants were first grown under fluctuating light (FL: 1 min 700 µmol photons m^{-2} s^{-1}, 4 min 70 µmol photons m^{-2} s^{-1}). For measurements under FL, plants grown for a minimum of 45 d (WT, *ggt2*) or 55 d (*ggt1* and *hpr1* lines) were taken directly out of the light and exposed to 45 min light fluctuations (as growth light), then 15 min of 200 µmol photons m^{-2} s^{-1}, and subsequently 30 min darkness. For measurements under control and low light, plants were grown for 39 d (WT and *ggt2*) or at least 50 d (*ggt1* and *hpr1* lines) under FL, allowing the mutants to accumulate sufficient leaf area for the measurement and then shifted to CL or LL for at least 5 days to allow for acclimation to the new light condition. Gas exchange was measured for 45 min at growth light intensity and subsequent 30 min darkness. Gas exchange was determined in air (21% O_2) or low oxygen (2% O_2). Net CO_2 assimilation rate (A) and internal CO_2 concentration C_i were averaged over the final 10 min of the FL regime and over 1.5 min in steady state of CL or LL. O_2-surpressed CO_2 assimilation rate (A_{sup}) of each plant was calculated as the difference in CO_2 assimilation rate under both gas environments. Two-way ANOVA and subsequent Tukey multiple comparisons were conducted with gas exchange parameters as dependent variable and genotype and growth light condition as factors. Significant differences with $p < 0.05$ are printed in bold and blue. Diff - Difference, P - number of means spanned in comparison.

Supplementary Data 2: Changes in levels of selected proteins during a shift from fluctuating to control light.
Quantification of immunoblot signals of total leaf protein from WT, *ggt2*, *ggt1-1* and *hpr1-1* grown under fluctuating light (FL: 1 min 700 µmol photons m^{-2} s^{-1}, 4 min 70 µmol photons m^{-2} s^{-1}; 35 d) and shifted to control light (CL: 200 µmol photons m^{-2} s^{-1}). Samples were taken before the light shift (35 d in FL) and 27 h (1 d CL) and 123 h (5 d CL) after the light shift. Membranes were stained with Ponceau Red (P.R.) and subsequently incubated with anti-GGT1/2, anti-HPR1 and anti-APX. Anti-GGT binds both GGT isoforms and anti-APX recognizes four isoforms of APX localized in the thylakoid (tAPX), stroma (sAPX) and in the peroxisome and cytosol (p+cAPX, indistinguishable in size). All membranes can be found in Supplementary Fig. 10. Immunoblot signals were normalized on the RbcL P.R. signals of each membrane (M) and WT levels from FL before the shift. Asterisks indicate significant differences between mutant and WT at a given time point and different lower-case letters indicate significant differences between protein levels within one genotype as determined by two-way ANOVA and subsequent Tukey multiple comparison test with $p < 0.05$. SD - Standard deviation

Supplementary Data 3: Changes in levels of selected proteins during a shift from control to fluctuating light.
Quantification of immunoblot signals of total leaf protein from WT, *ggt2*, *ggt1-2* and *hpr1-2* grown under control light (CL: 200 µmol photons m^{-2} s^{-1}; 29 d) and shifted to fluctuating light (FL: 1 min 700 µmol photons m^{-2} s^{-1}, 4 min 70 µmol photons m^{-2} s^{-1}). Samples were taken before the light shift (29 d in CL) and 27 h (1 d FL) and 123 h (5 d FL) after the light shift. Membranes were stained with Ponceau Red (P.R.) and subsequently incubated with anti-GGT1/2, anti-HPR1 and anti-APX. Anti-GGT binds both GGT isoforms and anti-APX recognizes four isoforms of APX localized in the thylakoid (tAPX), stroma (sAPX) and in the peroxisome and cytosol (p+cAPX, indistinguishable in size). All membranes can be found in Supplementary Fig. 10. Immunoblot signals were normalized on the RbcL P.R. signals of each membrane (M) and WT levels from CL before the shift. Asterisks indicate significant differences

between mutant and WT at a given time point and different lower-case letters indicate significant differences between protein levels within one genotype as determined by two-way ANOVA and subsequent Tukey multiple comparison test with $p < 0.05$. SD - Standard deviation

Supplementary Data 4: Averaged metabolite data during light shift and control experiments.
WT, *ggt2* and two mutant alleles of each *ggt1* and *hpr1* were grown under fluctuating light (FL: 1 min 700 µmol photons m^{-2} s^{-1}, 4 min 70 µmol photons m^{-2} s^{-1}) and control light (CL: 200 µmol photons m^{-2} s^{-1}) and either shifted to CL (FL to CL) or FL (CL to FL), respectively, or kept under one light condition continuously (FL and CL control). Shifts were performed 3 h into the light period. Samples were always taken 6 h into the light period, on the day before the light shift (day 35 for FL and 29 for CL), 3 h, 27 h, 75 h and 123 h after the shift. FL and CL controls were sampled at the same time points as the shifted plants. Averages of n = 3-4 ± standard deviation (SD) are shown.

Supplementary Data 5: Statistical analysis of differences in carbohydrates, amino acids, glutamate and glutamine during light shifts and control experiments.
Statistical analysis of total carbohydrate and free amino acid levels and selected metabolites. WT, *ggt2* and two mutant alleles of each *ggt1* and *hpr1* were grown under fluctuating light (FL: 1 min 700 µmol photons m^{-2} s^{-1}, 4 min 70 µmol photons m^{-2} s^{-1}) and control light (CL: 200 µmol photons m^{-2} s^{-1}) and either shifted to CL (FL to CL) or FL (CL to FL) or kept under one light condition continuously (FL and CL control). Shifts were performed 3 h into the light period. Samples were always taken 6 h into the light period, on the day before the light shift (day 35 for FL and 29 for CL), 3 h, 27 h, 75 h and 123 h after the shift. FL and CL controls were sampled at the same time points as the shifted plants. Asterisks indicate significant differences between mutant and WT at a given time point and different lower-case letters indicate significant differences between timepoints within one genotype for each light condition/shift as determined by two-way ANOVA and subsequent Tukey multiple comparison test with $p < 0.05$.

Supplementary Data 6: Predicted fluxes through selected reactions of photorespiration, the tricarboxylic acid cycle and NAD(P)H and ATP metabolism
The AraCoreTFA model was parametrized with CO2 assimilation rates, relative growth rates and metabolite concentrations to predict flux distributions for the Col-0 wild-type (WT) and photorespiratory mutants *ggt1-1*, *ggt1-2*, *hpr1-1*, *hpr1-2* in control light (CL: 200 µmol photons m^{-2} s^{-1}) and fluctuating light (FL: 1 min 700 µmol photons m^{-2} s^{-1}, 4 min 70 µmol photons m^{-2} s^{-1}) condition (see Material and Methods and SI Methods for a detailed description) as in Fig. 5. Flux through selected reactions associated with photorespiration, tricarboxylic acid (TCA) cycle, ATP and NAD(P)H metabolism are shown. The flux sum through a metabolite is one half of the sum of absolute value of the contributions of different reactions that alter the concentration of the metabolite. Letters in square brackets next to metabolites in reaction equations indicate localization of the metabolite with h - chloroplast, p - peroxisome, m - mitochondrion and c - cytosol. Rubisco - Ribulose biphosphate carboxylase/ oxygenase, RuBP - Ribulose-1,5-bisphosphate, 2PG - 2-phosphoglycolate, PGA - 3-phosphoglycerate, 2OG – 2-oxoglutarate, A-CoA - Acetyl-CoA, amDHP - Aminomethyldihydrolipoylprotein, cACN - cis-aconitate, Cit - Citrate, CO2 - Carbon dioxide, DHP - Dihydrolipolprotein, Fum - Fumarate, GCA – Glycolate, GCEA – Glycerate, Glu – Glutamate, GLX – Glyoxylate, Gly – Glycine, H - Hydrogen, H2O - Water, H2O2 - Hydrogen peroxide, HPR – Hydroxypyruvate, iCit - Isocitrate, LPA - Lipoamide, LPL - Lipoylprotein, Mal - Malate, M-THF - 5,10-

Methylenetetrahydrofolate, NH4 - Ammonium, O2 - Oxygen, OAA - Oxaloacetate, Q - Ubiquinone, QH2 - Ubiquinol, SCA - Succinate, S-CoA - Succinyl CoA, S-DHL - Succinyldihydrolipoamide, Ser - L-Serine, THF - 5,6,7,8-Tetrahydrofolate

Supplementary Data 7: Statistical analysis of reaction fluxes related to photorespiration and tricarboxylic acid cycle.

Pairwise comparisons of metabolites fluxes were performed between all genotypes (Col-0 (WT), *ggt1-1*, *ggt1-2*, *hpr1-1*, *hpr1-2*) within simulated light conditions (CL: control light, 200 µmol photons m^{-2} s^{-1}; FL: fluctuating light, 90 µmol photons m^{-2} s^{-1}) at the -21 h (t0) timepoint. The upper right triangles for the genotype by genotype matrices below contain p-values or effect sizes for the comparisons within the CL condition, while the lower left triangles show the results for the FL condition. Further, it was tested whether there is a difference in median values between the CL and FL conditions for each genotype, respectively ("CL vs FL"). For each statistical comparison, the Wilcoxon rank sum test was used and p-values were corrected for multiple testing using the Benjamini-Hochberg procedure. If the difference between two samples was below $2x10^{-9}$ (2x feasibility tolerance of the solver), the associated p-value was set to one, indicating no difference. The associated z statistics to compute the approximate p-values are provided in an additional column. Further, the 95% confidence intervals of the median flux differences are given. These were determined by bootstrapping the median flux difference with 1000 samples and calculating the 2.5% and 97.5% percentiles of the resulting distribution. The effect sizes were determined by calculating the Wilcox Q (Wilcox (2019), DOI: 10.22237/jmasm/1551905677) and transforming them by $\Omega = |(Q-0.5)| / 0.5$, such that Ω=0 indicates the lowest and Ω=1 indicates the highest effect size. If the maximum flux difference between two samples was smaller than $2x10^{-9}$, they were considered identical the and value for Ω was set to zero.

Supplementary Data 8: Data used to adjust the AraCoreTFA model to different light conditions and genotypes.

The experimentally-determined ratios between oxygenation and carboxylation fluxes, φ, were used to constrain the respective fluxes in the model. Light intensities were converted to upper limits for the photon uptake reaction in the model (v_{hv}^{max}) by scaling the default upper bound of 1000 mmol gDW^{-1} h^{-1} by the ratio between the light intensity (I) and the saturating light intensity of 700 µmol m^{-2} s^{-1}. Relative growth rates (RGR) were calculated following the equation $DW_t=DW_0 \cdot e^{RGR.t}$, where DW_t is the dry weight measured after t hours and DW_0 is the dry weight of the seeds. *A* is the measured net CO_2 assimilation rate.
