# Supplementary material for: Growth in fluctuating light buffers plants against photorespiratory perturbations
Source: Nat Commun. 2023 Nov 3;14:7052. doi: 10.1038/s41467-023-42648-x (PMC10624928; doi:10.1038/s41467-023-42648-x)
Supplement: Supplementary file 12 — Reporting Summary [file 41467_2023_42648_MOESM12_ESM.pdf]

nature portfolio

Corresponding author(s): Thekla von Bismarck
Ute Armbruster

Last updated by author(s): Oct 2, 2023

Reporting Summary

Nature Portfolio wishes to improve the reproducibility of the work that we publish. This form provides structure for consistency and transparency in reporting. For further information on Nature Portfolio policies, see our Editorial Policies and the Editorial Policy Checklist.

Statistics

For all statistical analyses, confirm that the following items are present in the figure legend, table legend, main text, or Methods section.

n/a	Confirmed	
☐	☒	The exact sample size (*n*) for each experimental group/condition, given as a discrete number and unit of measurement
☐	☒	A statement on whether measurements were taken from distinct samples or whether the same sample was measured repeatedly
☐	☒	The statistical test(s) used AND whether they are one- or two-sided<br>*Only common tests should be described solely by name; describe more complex techniques in the Methods section.*
☐	☒	A description of all covariates tested
☐	☒	A description of any assumptions or corrections, such as tests of normality and adjustment for multiple comparisons
☐	☒	A full description of the statistical parameters including central tendency (e.g. means) or other basic estimates (e.g. regression coefficient) AND variation (e.g. standard deviation) or associated estimates of uncertainty (e.g. confidence intervals)
☐	☒	For null hypothesis testing, the test statistic (e.g. *F*, *t*, *r*) with confidence intervals, effect sizes, degrees of freedom and *P* value noted<br>*Give P values as exact values whenever suitable.*
☒	☐	For Bayesian analysis, information on the choice of priors and Markov chain Monte Carlo settings
☒	☐	For hierarchical and complex designs, identification of the appropriate level for tests and full reporting of outcomes
☐	☒	Estimates of effect sizes (e.g. Cohen's *d*, Pearson's *r*), indicating how they were calculated

Our web collection on statistics for biologists contains articles on many of the points above.

Software and code

Policy information about availability of computer code

Data collection	All data was collected as described in the material and methods section. The code for thermodynamics-based flux analysis is available at GitHub (see data availability statement)
Data analysis	Statistical analysis was conducted using Matlab R2020b, SigmaPlot 14.5 or Python 3.7.4, as described in material and methods.

For manuscripts utilizing custom algorithms or software that are central to the research but not yet described in published literature, software must be made available to editors and reviewers. We strongly encourage code deposition in a community repository (e.g. GitHub). See the Nature Portfolio guidelines for submitting code & software for further information.

Data

Policy information about availability of data

All manuscripts must include a data availability statement. This statement should provide the following information, where applicable:

- Accession codes, unique identifiers, or web links for publicly available datasets
- A description of any restrictions on data availability
- For clinical datasets or third party data, please ensure that the statement adheres to our policy

Source data for Figs. 1-5 and Supplementary Figs. 3-7 & 9-14 are provided with the paper. All raw chromatograms for metabolomics analysis are publicly available as a MassIVE dataset MSV000091798 under the doi: 10.25345/C5377651Q [https://massive.ucsd.edu/ProteoSAFe/dataset.jsp?task=f103606327b84c0b84533de4d59592fe].

Research involving human participants, their data, or biological material

Policy information about studies with human participants or human data. See also policy information about sex, gender (identity/presentation), and sexual orientation and race, ethnicity and racism.

Reporting on sex and gender	*Use the terms sex (biological attribute) and gender (shaped by social and cultural circumstances) carefully in order to avoid confusing both terms. Indicate if findings apply to only one sex or gender; describe whether sex and gender were considered in study design; whether sex and/or gender was determined based on self-reporting or assigned and methods used. Provide in the source data disaggregated sex and gender data, where this information has been collected, and if consent has been obtained for sharing of individual-level data; provide overall numbers in this Reporting Summary. Please state if this information has not been collected. Report sex- and gender-based analyses where performed, justify reasons for lack of sex- and gender-based analysis.*
Reporting on race, ethnicity, or other socially relevant groupings	*Please specify the socially constructed or socially relevant categorization variable(s) used in your manuscript and explain why they were used. Please note that such variables should not be used as proxies for other socially constructed/relevant variables (for example, race or ethnicity should not be used as a proxy for socioeconomic status). Provide clear definitions of the relevant terms used, how they were provided (by the participants/respondents, the researchers, or third parties), and the method(s) used to classify people into the different categories (e.g. self-report, census or administrative data, social media data, etc.) Please provide details about how you controlled for confounding variables in your analyses.*
Population characteristics	*Describe the covariate-relevant population characteristics of the human research participants (e.g. age, genotypic information, past and current diagnosis and treatment categories). If you filled out the behavioural & social sciences study design questions and have nothing to add here, write "See above."*
Recruitment	*Describe how participants were recruited. Outline any potential self-selection bias or other biases that may be present and how these are likely to impact results.*
Ethics oversight	*Identify the organization(s) that approved the study protocol.*

Note that full information on the approval of the study protocol must also be provided in the manuscript.

Field-specific reporting

Please select the one below that is the best fit for your research. If you are not sure, read the appropriate sections before making your selection.

☒ Life sciences ☐ Behavioural & social sciences ☐ Ecological, evolutionary & environmental sciences

For a reference copy of the document with all sections, see nature.com/documents/nr-reporting-summary-flat.pdf

Life sciences study design

All studies must disclose on these points even when the disclosure is negative.

Sample size	Sample size was not statistically predetermined. For growth analysis and fluorescence measurements, a standard sample size of n = 7-10 was sufficient as the phenotypes were highly reproducible. For protein analysis, gas exchange measurements, and metabolomics analysis, the sample size was as big as feasible for our experimental approaches.
Data exclusions	Shown is average data described in the Materials and Methods. Raw data is provided in the supplementary tables, the source data or available online (see data availability statement). Exploratory results on test plants purely to establish the optimal instrument settings were not included in statistical analyses. Due to technical problems, some gas exchange measurements were repeated the following day on the same plant and the experimental replicate data was included in the analysis. Western Blot detecting APX were excluded, when signals for different APX isoforms were not separated sufficiently or if the signal was too low to be detected. All metabolite data was manually revised and therefore N.A.s correspond to metabolites below the limit of detection for the respective sample. For determination of total carbohydrates and free amino acids, replicates were excluded for which the main constituent (sucrose or serine and glutamine, respectively) could not be detected.
Replication	All growth measurements were highly reproducible, as evident by multiple independent experiments included in the study (see Supplementary Information). For fluorescence, protein and metabolite analysis, light shifts were always conducted in both directions and for the metabolomics analysis, samples of unshifted plants under both conditions served as an additional control.
Randomization	All plants were randomized in the growth chambers to avoid edge effects (variations in light, temperature and humidity). To avoid time-of-day effects, wild type and mutant lines were measured throughout the day in a randomized fashion.
Blinding	Planting, growth and pricking of plants were performed by gardeners. It was not possible to maintain blinding as some analysis required several labs to process and analyze the data in a consecutive manner (e.g., metabolomics) and blinding would have risked mislabeling of the samples.

Reporting for specific materials, systems and methods

We require information from authors about some types of materials, experimental systems and methods used in many studies. Here, indicate whether each material, system or method listed is relevant to your study. If you are not sure if a list item applies to your research, read the appropriate section before selecting a response.

Materials & experimental systems

n/a	Involved in the study	
☐	☒	Antibodies
☒	☐	Eukaryotic cell lines
☒	☐	Palaeontology and archaeology
☒	☐	Animals and other organisms
☒	☐	Clinical data
☒	☐	Dual use research of concern
☐	☒	Plants

Methods

n/a	Involved in the study	
☒	☐	ChIP-seq
☒	☐	Flow cytometry
☒	☐	MRI-based neuroimaging

Antibodies

Antibodies used	APX (Agrisera, AS08368), HPR1 (custom-made; first described in Timm, 2008), GGT1/2 (custom-made; first described in this study)
Validation	Validation of the HPR1 and GGT1/2 antibodies is included in the manuscript. They did not give any signal (HPR1) or strongly reduced signal (GGT1/2) at a molecular size corresponding to the detected protein in hpr1-1 and ggt1-1, respectively (Supplemental Fig S10)

Dual use research of concern

Policy information about dual use research of concern

Hazards

Could the accidental, deliberate or reckless misuse of agents or technologies generated in the work, or the application of information presented in the manuscript, pose a threat to:

No	Yes	
☒	☐	Public health
☒	☐	National security
☒	☐	Crops and/or livestock
☒	☐	Ecosystems
☒	☐	Any other significant area

Experiments of concern

Does the work involve any of these experiments of concern:

No	Yes	
☒	☐	Demonstrate how to render a vaccine ineffective
☒	☐	Confer resistance to therapeutically useful antibiotics or antiviral agents
☒	☐	Enhance the virulence of a pathogen or render a nonpathogen virulent
☒	☐	Increase transmissibility of a pathogen
☒	☐	Alter the host range of a pathogen
☒	☐	Enable evasion of diagnostic/detection modalities
☒	☐	Enable the weaponization of a biological agent or toxin
☒	☐	Any other potentially harmful combination of experiments and agents
